# *N*-Acyl-*N*-Alkyl Sulfonamide Probes for Ligand-Directed Covalent Labeling of GPCRs: The Adenosine A_2B_ Receptor as Case Study

**DOI:** 10.1021/acschembio.4c00210

**Published:** 2024-06-26

**Authors:** Bert L. H. Beerkens, Vasiliki Andrianopoulou, Xuesong Wang, Rongfang Liu, Gerard J. P. van Westen, Willem Jespers, Adriaan P. IJzerman, Laura H. Heitman, Daan van der Es

**Affiliations:** † Division of Medicinal Chemistry, Leiden Academic Centre for Drug Research, Leiden University, Einsteinweg 55, 2333 CC Leiden, The Netherlands; ‡ Oncode Institute, Einsteinweg 55, 2333 CC Leiden, The Netherlands

## Abstract

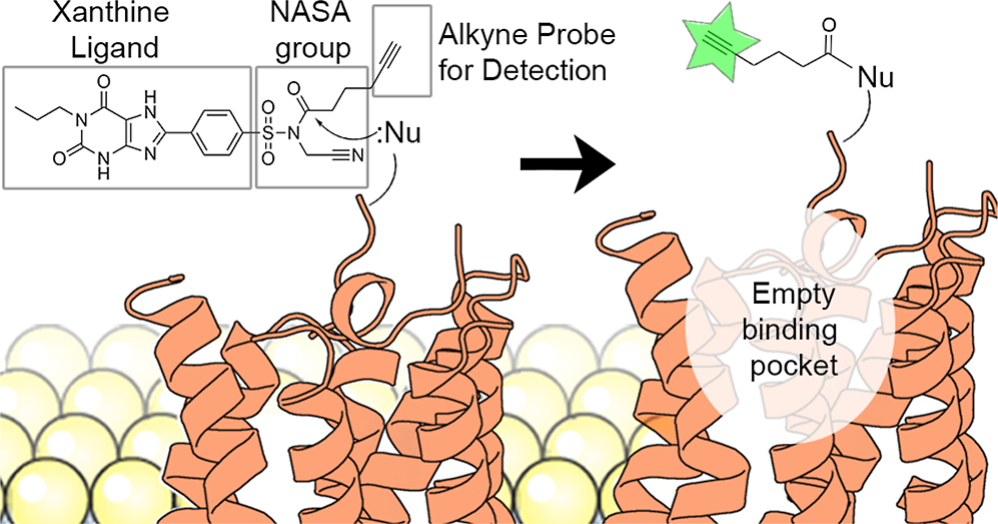

Small molecular tool compounds play an essential role in the study of G protein-coupled receptors (GPCRs). However, tool compounds most often occupy the orthosteric binding site, hampering the study of GPCRs upon ligand binding. To overcome this problem, ligand-directed labeling techniques have been developed that leave a reporter group covalently bound to the GPCR, while allowing subsequent orthosteric ligands to bind. In this work, we applied such a labeling strategy to the adenosine A_2B_ receptor (A_2B_AR). We have synthetically implemented the recently reported *N*-acyl-*N*-alkyl sulfonamide (NASA) warhead into a previously developed ligand and show that the binding of the A_2B_AR is not restricted by NASA incorporation. Furthermore, we have investigated ligand-directed labeling of the A_2B_AR using SDS-PAGE, flow cytometric, and mass spectrometry techniques. We have found one of the synthesized probes to specifically label the A_2B_AR, although detection was hindered by nonspecific protein labeling most likely due to the intrinsic reactivity of the NASA warhead. Altogether, this work aids the future development of ligand-directed probes for the detection of GPCRs.

## Introduction

G protein-coupled receptors (GPCRs) are a family of membrane proteins that are involved in many pathological and physiological processes. Hence, GPCRs have been a major target in drug discovery programs, yielding countless ligands and multiple approved clinical candidates.^1,2^ The adenosine A_2B_ receptor (A_2B_AR), an exemplary GPCR, has recently started attracting attention due to its immunosuppressive role in the tumor microenvironment.^3−5^ Antagonizing the A_2B_AR is therefore an interesting new strategy in immuno-oncology.

To study all aspects of GPCR signaling, e.g. expression levels, intracellular pathways or ligand binding kinetics, tool compounds play an important role. An advantage of small molecular tool compounds as compared to genetically encoded receptors (e.g. containing a GFP-, BRET- or FRET-tag) is the opportunity to detect endogenously expressed receptors on cells and tissue-derived materials. Over the past decades, a wide variety of tool compounds has been developed to selectively target and detect GPCRs. These include fluorescent ligands, covalent ligands and affinity-based probes, among other types of probe molecules.^6−8^ In case of the A_2B_AR, there has been a recent surge in the development of chemical tools, i.e. pet tracers,^9−11^ fluorescent ligands,^12−14^ and covalent ligands.^15,16^ These types of tool compounds, however, all occupy the orthosteric binding site of GPCR, hampering the study of GPCR signaling upon ligand-induced activation.

**Figure 1:**
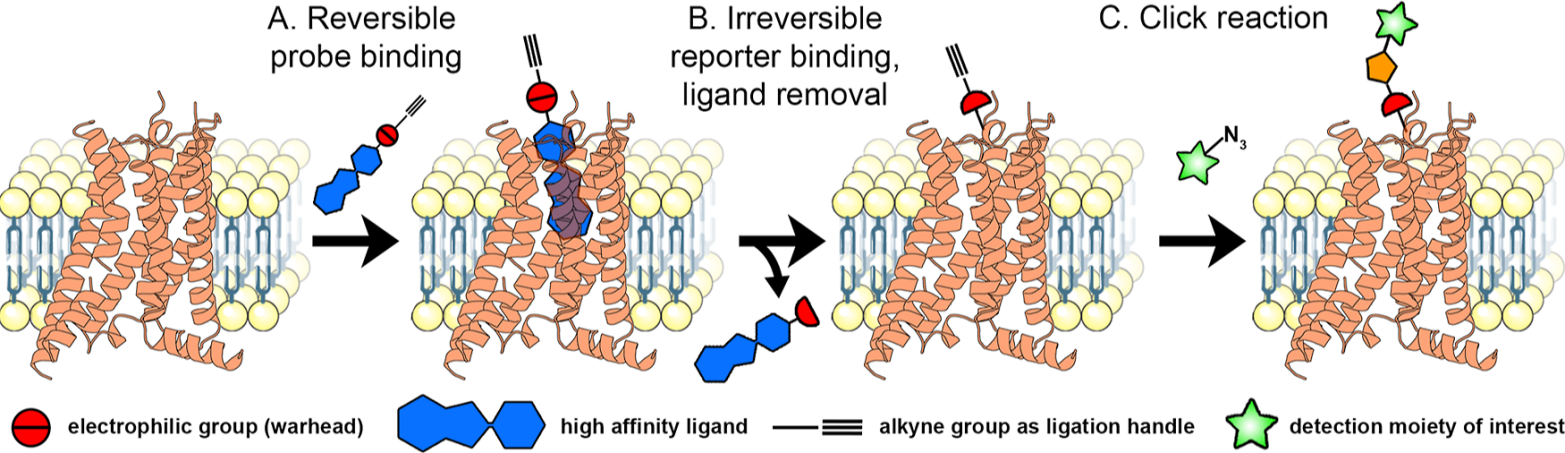
Schematic overview of the ligand-directed labeling of GPCRs. (A) The probe binds to the receptor through its conjugated high-affinity ligand. (B) A nucleophilic amino acid residue attacks the electrophilic group of the probe, inducing cleavage between the ligand and the reporter. The reporter group, in this case an alkyne group as click handle, is now covalently bound to the receptor, while the ligand is allowed to leave the binding pocket (reversible mode of binding). (C) The substituted alkyne group can be further derivatized by performing click chemistry using azide-containing detection moieties. This figure was partly created with Protein Imager,^28^ using the structure of the A_2B_AR (PDB: 8HDO).^29^

To overcome this problem, ligand-directed labeling techniques have been developed that covalently substitute a reporter group, such as a fluorophore, click handle or biotin group, onto the target protein.^17−19^ In brief, a high affinity ligand is conjugated to a cleavable electrophile and a desired reporter group. Upon binding of the ligand to the target protein, a nucleophilic amino acid residue in close proximity will attack the electrophile, leading to cleavage of the molecule and substitution of the reporter group with the protein (Figure 1A,B). In turn, the high-affinity ligand will be reversibly bound and thus able to leave the binding pocket, allowing tracing of the target protein upon activation by different sets of ligands.

Crucial for ligand-directed chemistry is the choice of the electrophile. A valid electrophile should not only induce bond cleavage between the high-affinity ligand and the reporter group but also bear a balanced reactivity: the electrophile has to react with an often poorly nucleophilic amino acid residue, driven by the induced proximity of the high-affinity ligand, but not react with any other amino acids within the proteome. Multiple electrophilic groups have been reported for their use in ligand-directed chemistries, such as tosyl,^17,20^ dibromophenyl benzoate,^8,21^ acyl imidazole,^18,22^ and *N*-acyl-*N*-alkyl sulfonamides.^19,23^ The *N*-acyl-*N*-alkyl sulfonamide (NASA) group in particular struck our interest as this group can be synthesized from fluorosulfonyl groups in a straightforward manner.

Our laboratory has previously reported on multiple fluorosulfonyl-containing GPCR ligands,^15,24,25^ allowing good starting points for the synthesis of GPCR ligands bearing the NASA group. From these ligands, we have chosen **1** (LUF7982), a covalent antagonist for the A_2B_AR,^15^ as a case study for the development of NASA-containing “ligand-directed probes”. In this work, we show the development of the first ligand-directed probes for the A_2B_AR. We report on the synthesis of two probe molecules, evaluate their binding toward the A_2B_AR, and use SDS-PAGE, flow cytometric, and mass spectrometry techniques to investigate whether the developed ligand-directed probes allow us to detect the A_2B_AR on live cells.

## Results and Discussion

### Design and Synthesis of A_2B_AR-Targeting Ligand-Directed Probes

**Scheme 1:**
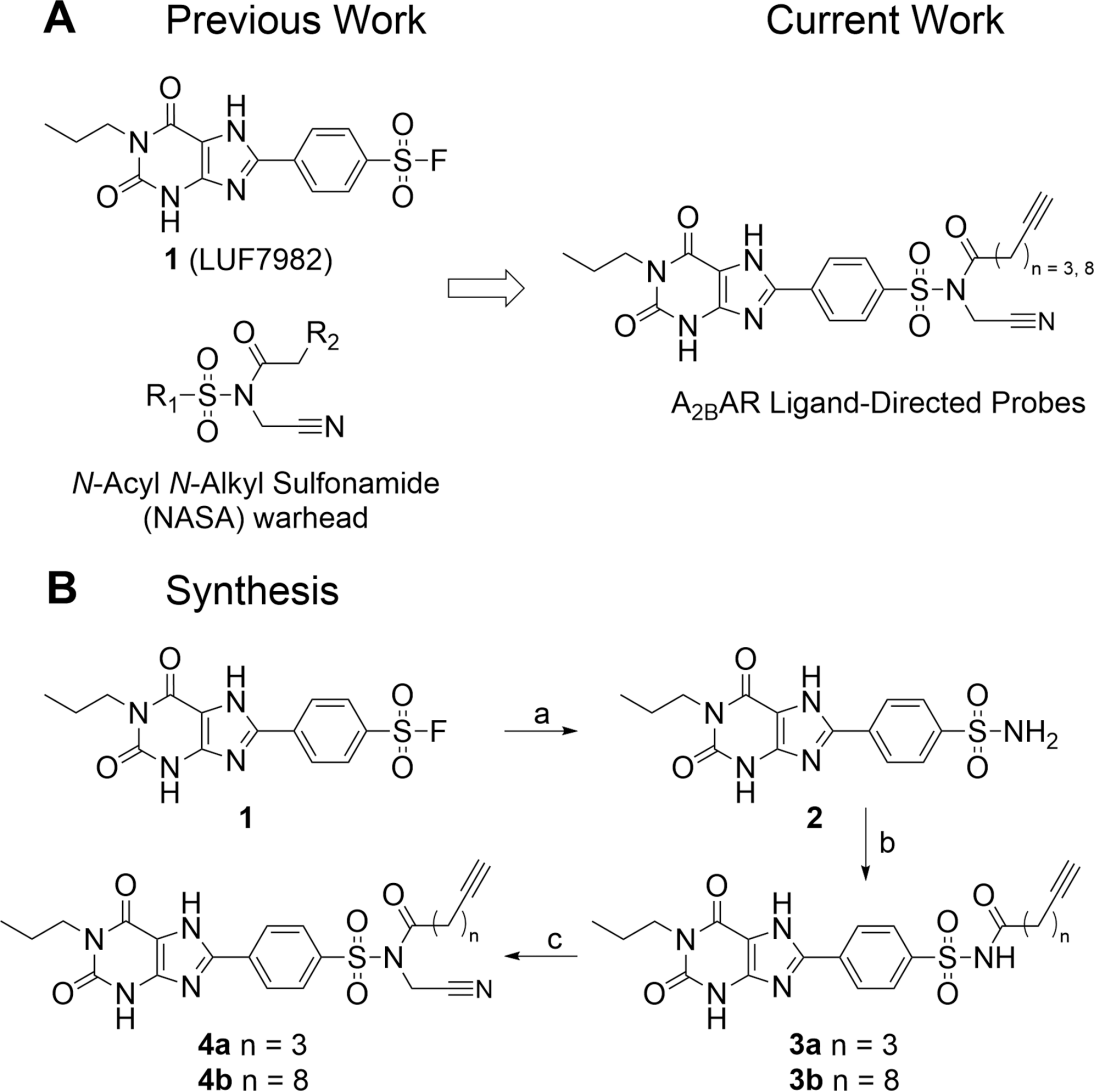
(A) Molecular Structures of the Previously Synthesized Covalent A_2B_AR Antagonist **1** (LUF7982), the NASA Warhead, and the Design of the Ligand-Directed Probes; R_1_ = Location of the Molecular Scaffold of a High-Affinity Ligand; R_2_ = Location of the Reporter Group for Detection; (B) Synthetic Scheme Towards Probes **4a** and **4b** Reagents and conditions. (a) NH_4_OH (28–30%), RT, 2 h, 75%; (b) EDC·HCl, respective benzoic acid, DMAP, DIPEA, dry DMF, RT, overnight, 41–64%; (c) bromoacetonitrile, DIPEA, RT, 6–8 days, 12–41%.

We and others have previously reported on xanthine-based compound **1** (LUF7982) that covalently binds to the A_2B_AR.^15,16^ While it was presumed that the attached fluorosulfonyl group forms a covalent bond with lysine residue 269 (K269) near the ligand binding pocket, site-directed mutagenesis studies did not prevent irreversible binding to the A_2B_AR, potentially hinting toward the involvement of residue lysine residue 267 (K267).^16^ Nonetheless, the location of the fluorosulfonyl group on the xanthine scaffold is a valid position for the implementation of an electrophilic group for ligand-directed chemistry. Like the fluorosulfonyl group, the NASA electrophile contains a sulfonyl moiety. Therefore, we envisioned that transforming the sulfonyl group of **1** into a NASA group would yield the first candidate A_2B_AR ligand-directed probes (Scheme 1A). To increase the electrophilicity of the acyl group, Tamura et al. substituted various electron-withdrawing groups onto the sulfonamide moiety, of which the cyano group showed to be superior in terms of reaction kinetics.^19^ Therefore, we also incorporated a cyano group into the design of our A_2B_AR-targeting probes. Next to that, we chose an alkyne group as reporter moiety, allowing the usage of copper-catalyzed click chemistry to “click” any reporter group of interest onto the acylated receptor (Figure 1C),^26,27^ without having to incorporate a bulky fluorophore in the design of the ligand. Lastly, we varied the length of the alkyl linker between the NASA and the alkyne group, as linker length might influence the affinity, reactivity, and stability of the compounds. We have therefore synthesized probes containing either a “short” 3-carbon linker or a “long” 8-carbon linker.

Synthesis of the two ligand-directed probes started with covalent antagonist **1**, synthesized as reported previously.^15^ First, the fluorosulfonyl group was transformed into a sulfonamide group using aqueous ammonium hydroxide (Scheme 1B). Sulfonamide **2** was coupled to either 5-hexynoic acid or 10-undecynoic acid using EDC·HCl, DMAP, and DIPEA to yield sulfonamides **3a** and **3b**. Lastly, the cyano moiety was introduced. Iodoacetonitrile, as used in other syntheses,^19^ showed to be too reactive for this step, resulting in oversubstitution at the secondary amines of **3a** and **3b**. Therefore, the milder bromoacetonitrile was used, yielding ligand-directed probes **4a** and **4b** as confirmed by NMR (^1^H, ^13^C, and HMBC) and HRMS.

### Affinity of Ligand-Directed Probes toward the A_2B_AR

First, to investigate the ability of the synthesized probes to bind to A_2B_AR, radioligand displacement assays were carried out. Control compounds **3a** and **3b**, lacking the cyano moiety, were included in these assays, and a concentration range from 0.1 to 1000 nM of the probe was chosen. Two different conditions were investigated: with and without 4 h of pre-incubation between A_2B_AR and ligand, prior to the addition of radioligand. Using this assay setup, we have previously observed that a time-dependent increase in affinity reflects the irreversible mode of binding of covalent ligands.^15^ All synthesized compounds showed a decent to good affinity toward the A_2B_AR, ranging from submicromolar (**3a**) to double- (**3b**, **4a**) and single-(**4b**) digit nanomolar values when not pre-incubated with receptor prior to the addition of radioligand (pre-0 h; Table 1). Of the four ligands, only **4b** showed to bind with a similar strong affinity as reference compound **1**. However, contrary to covalent antagonist **1**, none of the synthesized ligands showed a significant time-dependent increase in affinity upon 4 h of pre-incubation (pre-4 h; Table 1). This corresponds to the concept of ligand-directed labeling, in which the high-affinity ligand leaves the binding pocket upon a covalent donation of the reporter group. Interestingly, compound **4b** showed a slight decrease in affinity upon 4 h of pre-incubation. There are multiple possible explanations for this, for instance, the donated acyl group might influence the binding of other ligands to the A_2B_AR, as has been observed in a recent study on the adenosine A_2A_ receptor.^30^ Next, the subtype selectivity of the synthesized probes was investigated by single concentration (1 μM) radioligand displacement experiments on the other adenosine receptors (Table S1). Most of the synthesized compounds showed poor binding to the other ARs (<50% displacement), while only control compound **3b** showed a high displacement (82% at 1 μM radioligand) at the adenosine A_1_ receptor. Hence, ligand-directed probes **4a** and **4b** showed both a high affinity and good subtype selectivity toward the A_2B_AR.

**Table 1:** Time-dependent Affinity Values of the Synthesized Compounds Towards the A_2B_AR

Compd.	p*K*_i_ (pre-0 h)^a^	p*K*_i_ (pre-4 h)^b^	fold change^c^
**1**^d^	8.10 ± 0.06	9.17 ± 0.12**	12.1
**3a**	6.44 ± 0.06	6.60 ± 0.10	1.6
**3b**	7.26 ± 0.07	7.51 ± 0.06	1.8
**4a**	7.31 ± 0.05	7.44 ± 0.12	1.6
**4b**	8.22 ± 0.10	7.75 ± 0.08*	0.2

a Apparent affinity determined from the displacement of specific [^3^H]PSB-603 binding on CHO cell membranes stably expressing the A_2B_AR at 25 °C after 0.5 h of co-incubating probe and radioligand.

b Apparent affinity determined from the displacement of specific [^3^H]PSB-603 binding on CHO cell membranes stably expressing the A_2B_AR at 25 °C after 4 h of pre-incubation with the respective probe, followed by an additional 0.5 h of co-incubation with radioligand.

c Fold change determined by ratio *K*_i_(0 h)/*K*_i_(4 h).

d Values obtained from previous experiments.^15^ Data represent the mean ± SEM of three individual experiments performed in duplicate. *p < 0.05, **p < 0.01 compared to the p*K*_i_ values at pre-0 h, determined by a two-tailed unpaired Student’s *t*-test.

### Activation of the A_2B_AR after Probe Binding

**Figure 2:**
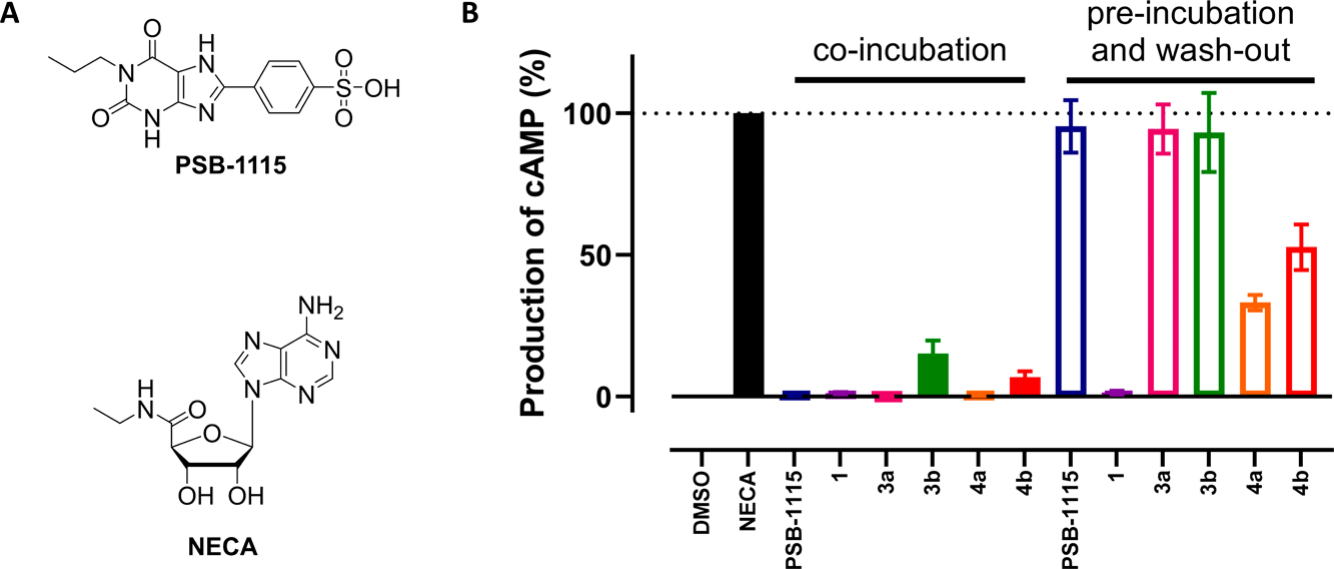
(A) Molecular structures of the reference agonist NECA and the reference antagonist PSB-1115. (B) Functional wash-out experiments. CHO-A_2B_AR cells were incubated with reversible antagonist PSB-1115, irreversible antagonist **1**, inactive probe **3a**, inactive probe **3b**, active probe **4a**, active probe **4b** or 0.1% DMSO (vehicle control in case of “DMSO” and “NECA”). The A_2B_AR was activated upon incubation with 100 nM of the agonist NECA, either through co-incubation with the respective ligand (“co-incubation”), or after pre-incubation with the respective ligands, followed by subsequent washing steps (“pre-incubation and wash-out”). DMSO (0.1%) was used as vehicle control for the NECA stimulation. Data represent the mean ± SEM of three individual experiments.

To investigate whether the A_2B_AR could still be activated after labeling, we performed functional assays that measure cAMP production upon agonist-induced activation of the A_2B_AR. CHO cells that stably express the A_2B_AR (CHO-A_2B_AR) were incubated with 10x the apparent *K*_i_ (pre-0 h; Table 1) of the structurally similar reversible antagonist PSB-1115 (Figure 2A),^31^ covalent antagonist **1**, control compounds **3a** or **3b**, or probes **4a** or **4b**. An incubation time of 30 min was chosen to prevent internalization of the A_2B_AR,^32^ but was also deemed appropriate for reversible binding of the probes (Table 1) and reactivity of the NASA group as determined by LC–MS experiments (Figure S1). The cells were either co-incubated with compound and the agonist NECA (“co-incubation”) or pre-incubated with compound, prior to multiple washing steps and stimulation with NECA (“pre-incubation and wash-out”). All the investigated compounds inhibited NECA-induced cAMP production when co-incubated with NECA, showing that the newly synthesized compounds all act as antagonists (Figure 2B). However, pre-incubation with the compounds and subsequent washing of the cells prior to NECA stimulation yielded a different outcome. Reversible antagonist PSB-1115 and control compounds **3a** and **3b** showed full recovery of the NECA-induced signal, indicating a reversible mode of binding, while covalent antagonist **1** retained full inhibition of signaling, indicating a covalent mode of action. The NECA-induced cAMP production of ligand-directed probes **4a** and **4b** is somewhere in between, indicating reduced activation of the A_2B_AR. Although probes **4a** and **4b** do not permanently block the ligand binding pocket, it seems that agonist-induced activation pathways are still hampered by the covalent substitution of the acyl group, resulting in a decreased cAMP production and thus a decreased A_2B_AR activity postlabeling.

Upon docking of the synthesized compounds (**4a** and **4b**) in an A_2B_AR model, we observed the electrophilic carbonyl group to be in close proximity to K269, much like parent compound **1**, suggesting a similar anchor point (Figure S2). Looking at the predicted binding modes, acylation of either K269 or K267 might hinder agonist entry to the ligand binding pocket, resulting in the observed reduction in activity.^30^

To further investigate the acylation of the receptor, we performed SDS-PAGE experiments.

### Labeling of Proteins in SDS-PAGE Experiments

**Figure 3:**
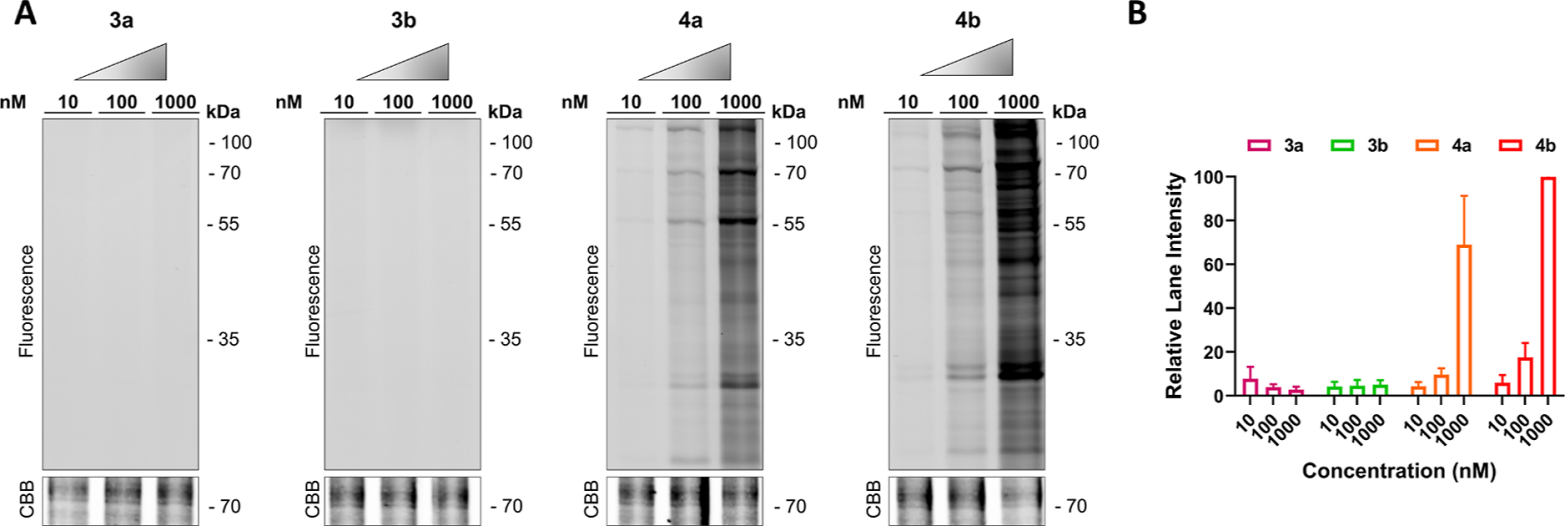
Ligand-directed labeling of the respective probes in CHO-A_2B_AR membrane fractions. CHO-A_2B_AR membrane fractions were incubated for 2 h with various concentrations of **3a**, **3b**, **4a**, or **4b**. Probe-bound proteins were clicked to Cy5–N_3_, denatured, and resolved by SDS-PAGE. (A) Gel images as taken using in-gel fluorescence. Coomassie Brilliant Blue (CBB) staining was used as a loading control. (B) Quantification of the lane intensities. The lane intensities were taken and corrected for the observed amount of protein per lane upon Coomassie staining. The lane intensity of 1000 nM **4b** was set to 100% and the other lanes were normalized accordingly. The mean values ± SEM of three individual experiments are shown.

In an initial screen, compounds **4a** and **4b**, as well as control compounds **3a** and **3b** were investigated for their general reactivity toward proteins in membrane fractions derived from CHO-A_2B_AR cells. The membrane fractions were incubated for 2 h with 10, 100, or 1000 nM of the respective probes, clicked to a cyanine-5 (Cy5) fluorophore, denatured, and resolved by SDS-PAGE. Ligand-directed probes **4a** and **4b** labeled multiple proteins at concentrations ≥ 10 nM (Figure 3A), while no concentration-dependent increase in labeling was observed for control compounds **3a** and **3b** (Figure 3B), indicating that the cyano substitution is necessary to enhance the electrophilicity of the *N*-acyl group. In previous experiments on the adenosine A_1_ and A_3_ receptors, we also observed labeling of multiple proteins upon using electrophilic probes in membrane-derived samples, presumably due to a combination of the electrophilic nature of the warhead with the high overall density of proteins (1 mg mL^–1^) used in the experiments.^33,34^ In these experiments, clearer labeling of the target GPCR was achieved when the experiment was performed on live cells. We therefore moved toward cellular assays in an attempt to observe more pronounced labeling of the A_2B_AR.

**Figure 4:**
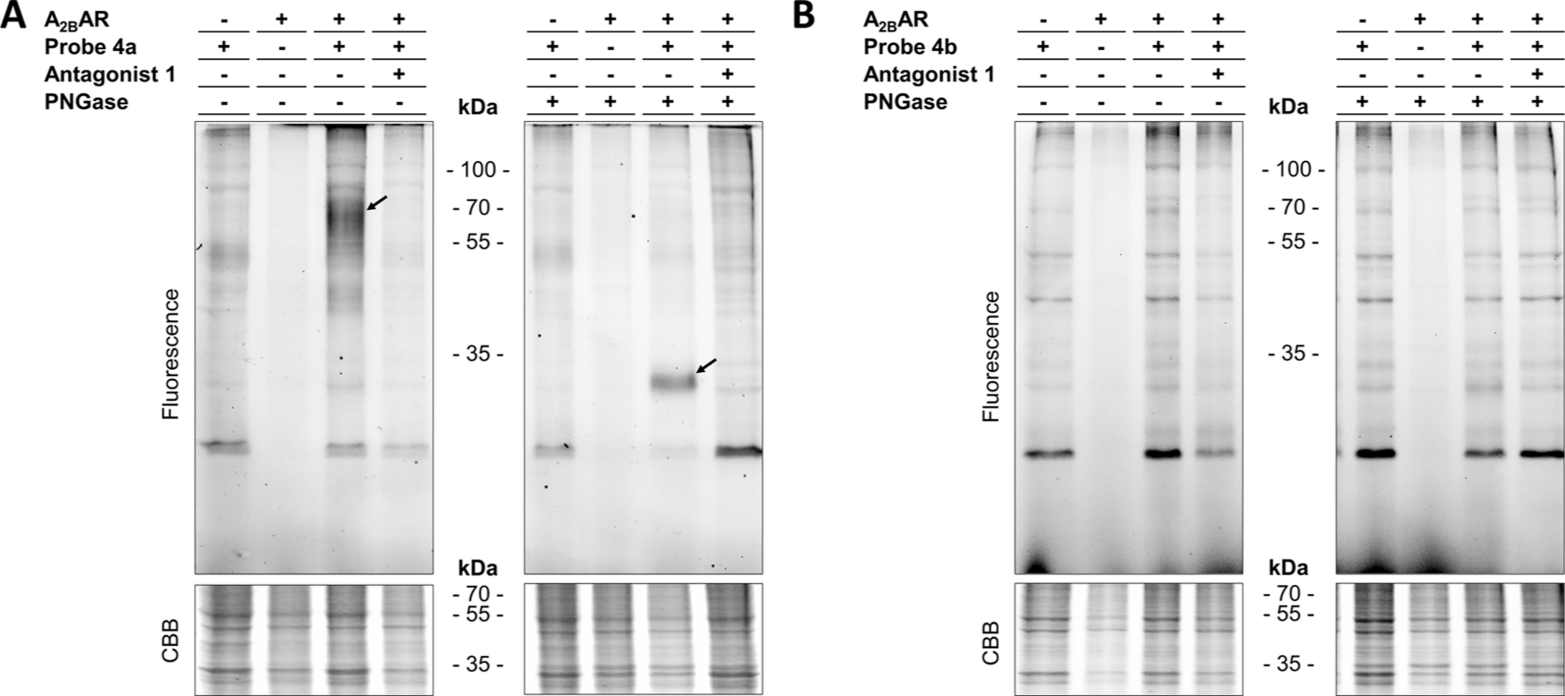
Ligand-directed labeling of A_2B_AR in live CHO cells. CHO cells with or without stable expression of the A_2B_AR were pre-incubated for 30 min with medium containing either 1% DMSO (vehicle) or 10 μM of irreversible antagonist **1**. The cells were subsequently incubated for 30 min with 400 nM probe in Hank’s Balanced Salt Solution (HBSS) or 1% DMSO (vehicle control). Cells were washed with PBS and membranes were collected. *N*-glycans were removed using PNGase (5 U) and alkyne moieties were clicked to 1 μM Cy5–N_3_. The samples were then denatured using Laemmli buffer and resolved by SDS-PAGE. Gels were imaged by in-gel fluorescence. CBB staining was used as the loading control. (A) Protein labeling by **4a**. The arrows indicate the presumable band of the A_2B_AR. (B) Protein labeling by **4b**. Gels are representatives of three replicates.

Live CHO cells with and without stable expression of the A_2B_AR were first pre-incubated with or without 10 μM of competing antagonist **1** and then incubated for 30 min with 400 nM (approximately 10 × *K*_i_) of ligand-directed probe **4a** or **4b**. The non-bound probe was washed away, and membrane fractions were collected. Probe-bound proteins were clicked to Cy5–N_3_, samples were denatured and loaded on SDS-PAGE, and the gels were visualized using in-gel fluorescence. Probe **4a** showed clear labeling of multiple proteins, but most interestingly is the smear at about 60 kDa (Figure 4A). This protein band was absent in the control lanes (without A_2B_AR, without probe or pre-incubation with covalent antagonist **1**) and therefore presumably belonged to the A_2B_AR. Removal of *N*-glycans through incubation with an excess of PNGase resulted in a disappearance of the smear at 60 kDa, while another more narrow band appeared at approximately 30 kDa. A similar pattern of bands has also been observed before in Western blot experiments and was characterized as being the A_2B_AR.^35−37^ Contrary to **4a**, compound **4b** did not show specific labeling of the A_2B_AR (Figure 4B), even after treatment with PNGase or using an incubation time of 2 h (Figure S3). Probe **4a** therefore seemed to be the best candidate for further labeling experiments.

### Live Cell Protein Labeling by Ligand-Directed Probes **4a** and **4b**

**Figure 5:**
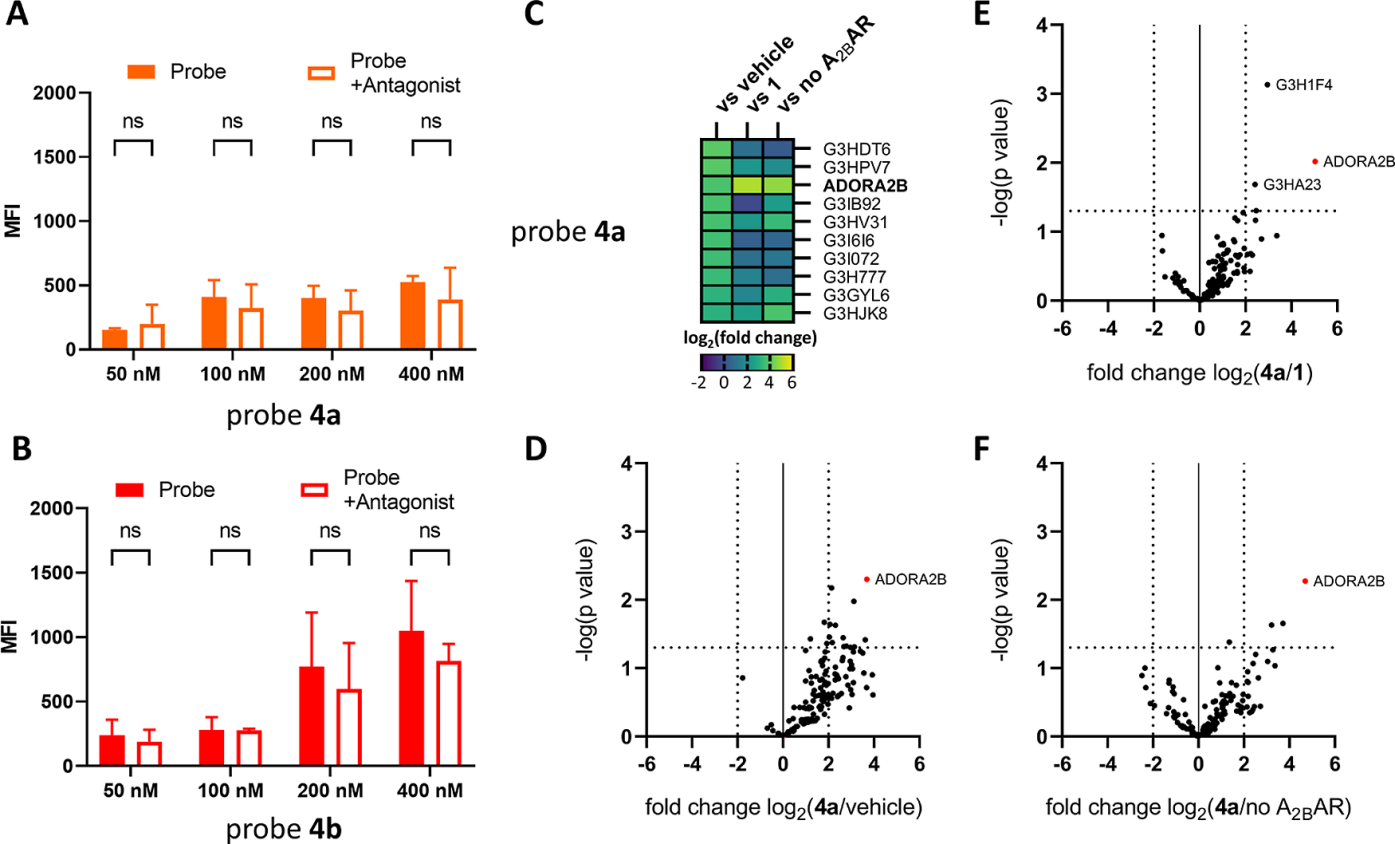
Labeling by probes **4a** and **4b** in live cell experiments. (A,B) Flow cytometry experiments. CHO cells stably overexpressing the A_2B_AR were pre-incubated for 30 min with 1% DMSO (vehicle) or irreversible antagonist **1** in medium, prior to incubation for 30 min with probe **4a** or **4b** in HBSS or 1% DMSO (vehicle control). The cells were fixed, permeabilized, clicked to Cy5–N_3_, and washed. The cells were then analyzed on their mean fluorescence intensity (MFI) by flow cytometry. Baseline correction was performed by subtraction of the MFI values by the average MFI value of the vehicle-treated samples. Shown are the mean values ± SEM of three individual experiments. ns = not significant. (C–F) Proteomic pull-down experiments using probe **4a**. Shown are log_2_(fold changes) depicting the intensity scores of probe-labeled proteins in positive samples, divided by the intensity scores of probe-labeled proteins in various control samples (vehicle, pre-incubation with antagonist **1** and without expression of the A_2B_AR). Fold changes are depicted by color in the case of the heat map (C) and on the *x*-axis of the volcano plots (D–F). CHO cells with or without (“no A_2B_AR”) stable expression of the A_2B_AR were pre-incubated for 30 min with a medium containing either 1% DMSO (vehicle) or 10 μM of irreversible antagonist **1** (“**1**”). The cells were subsequently incubated for 30 min with 400 nM probe in HBSS (“**4a**”) or 1% DMSO (vehicle control; “vehicle”). Cells were washed with PBS and membranes were collected. Alkyne moieties were clicked to Biotin–N_3_, reduced, alkylated, and pulled down using avidin beads. Bound proteins were digested into peptides, desalted, and analyzed by LC–MS/MS. (C) Heat map of the proteins labeled by probe **4a**. Shown are the top 10 proteins that showed the highest fold change over the vehicle. (D–F) Volcano plot comparing the **4a** labeled proteins toward various control conditions: vehicle, pre-incubation with antagonist **1** and CHO cells without expression of the A_2B_AR. Fold change is depicted on the *x*-axis and −log(*p* value) on the *y*-axis. Protein IDs (CHO proteins) and gene names (human proteins) are given and taken from UniProt. Data originate from three replicates.

Moving one step closer toward live cell labeling of the A_2B_AR, we carried out flow cytometry experiments as a more high-throughput technique to optimize labeling conditions. CHO-A_2B_AR cells were pre-incubated either with or without competing antagonists, incubated with probe **4a** or **4b**, washed, fixed, clicked to Cy5–N_3_, and scanned on fluorescence per cell. Within these assays, we aimed at obtaining a “window” of fluorescence that could be specifically attributed to the A_2B_AR, i.e., a difference in fluorescent intensity between the cells that were and were not pretreated with an excess of covalent antagonist **1**. The conditions that yielded A_2B_AR labeling in SDS-PAGE experiments did not yield a window of specific A_2B_AR fluorescence in flow cytometry experiments. We therefore lowered the probe concentration in an attempt to prevent off-target reactivity; however, still no significant difference was observed between cells that were and were not pretreated with a high concentration of **1** (Figure 5A,B). We further investigated different competing ligands, probe incubation times, fluorophore types, and fluorophore concentrations, but none of these alterations yielded a window of specific A_2B_AR fluorescence. As probes **4a** and **4b** also showed to be highly reactive toward proteins in standard cellular medium (Figure S1), we hypothesized that the NASA electrophile might be subject to unwanted side reactions during flow cytometry experiments. Such side reactions might be promoted by blocking the binding site of A_2B_AR, as observed in the SDS-PAGE experiments (Figure 4).

To further investigate protein labeling on live cells, we performed pull-down experiments using a biotin click and avidin beads. Similar to the SDS-PAGE experiments, CHO-A_2B_AR cells were incubated with probe **4a** or **4b**. Membranes were then collected, probe-bound proteins were clicked to biotin-N_3_, pulled down by avidin beads, digested using chymotrypsin, and measured by LC–MS/MS. The signal intensities of the probe-bound proteins were divided by their respective signal intensities in various control samples, yielding the indicated fold changes (Figure 5C–F). In case of probe **4a**, the A_2B_AR was enriched by the protein pull-down as one peptide of the receptor (SHANSVVNPIVY) showed up with a high fold change (>4) compared to the various controls (vehicle, pre-incubation with **1** and no A_2B_AR), indicating that the A_2B_AR is not only bound but also labeled by probe **4a** (Figure 5C–F). The large number of other proteins that also exhibited a positive fold change compared to one of the controls indicates a broader reactivity of probe **4a**. Most of these off-target proteins were not significantly enriched (Figure 5D–F) and therefore presumably either false positives or the result of nonselective labeling. The proteins that, besides the A_2B_AR, showed a reduction in signal intensity upon pre-incubation with antagonist **1** were the ribosomal protein G3H1F4 and malate dehydrogenase G3HA23 (Figure 5E). These two proteins were also present in the vehicle control samples and therefore presumably background proteins. Antagonist **1** thus selectively inhibits labeling of the A_2B_AR by probe **4a**, indicating the preference of the used xanthine-based scaffold for the A_2B_AR over other proteins. In case of probe **4b**, fewer proteins were detected in pull-down experiments, of which none showed a reduction upon pretreatment with antagonist **1** (Figure S4). This corresponds to the lack of A_2B_AR labeling by **4b** as seen in the SDS-PAGE experiments (Figure 4B). Taken together, the observed off-target protein labeling by probes **4a** and **4b** does not seem to be caused by the binding of the xanthine scaffold to a specific set of proteins and is therefore presumably caused by the high reactivity of the NASA group. This corresponds to the lack of decrease in fluorescent labeling observed in the flow cytometry experiments.

## Conclusions

Over the past few years, the development of tool compounds making use of ligand-directed labeling techniques (“ligand-directed probes”) has gained interest within the field of GPCR research. Multiple GPCR-targeting ligand-directed probes have been developed, e.g., for de bradykinin B_2_ receptor (B_2_R),^38^ adenosine A_2A_ receptor (A_2A_AR),^30,39^ μ opioid receptor (MOR),^22^ cannabinoid receptor type 2 (CB_2_R),^23^ and smoothened receptor.^40^ Most interesting are applications that allow labeling and tracing of target GPCRs in endogenous cells and tissues, such as the use of an A_2A_AR ligand-directed probe in breast cancer cell lines,^39^ and a MOR ligand-directed probe in rodent brain slices.^22^

In this work, we have examined the NASA group as a potential electrophile for ligand-directed labeling of GPCRs. By converting the fluorosulfonyl group of a known A_2B_AR ligand into the NASA warhead, we managed to label and detect the A_2B_AR in multiple assay types, such as SDS-PAGE and chemical proteomics. However, the detection of the A_2B_AR seems to be hampered due to nonspecific labeling. Previous studies have reported on the selective labeling of target proteins using NASA-containing ligands, e.g., the folate receptor and the CB_2_R.^19,23^ Although “overexpressed”, the concentration of the A_2B_AR in the herein used CHO-A_2B_AR membranes is approximately 4.30 pmol/mg.^41^ Such concentrations of protein cause a decrease in signal-to-noise ratio and therefore a higher prevalence of non-selectively labeled proteins. In previous studies using the fluorosulfonyl warhead, a less reactive electrophile,^42^ we also observed that expression levels greatly influence the detection of labeled GPCRs.^33,34^ Therefore, the NASA electrophile might not have the right “balanced” reactivity to selectively label GPCRs at low expression levels, which is the case for most endogenous GPCRs.

While writing this manuscript, Hamachi et al., who developed the first NASA-containing ligands, reported on the limitations of the NASA electrophile due to the high intrinsic reactivity.^42^ These findings correspond to the reactivity of the herein synthesized probes **4a** and **4b**, as observed in LC–MS (Figure S1), flow cytometry (Figure 5A,B) and pull-down (Figure 5C–F) experiments. The authors therefore reported on the second generation of NASA warheads, in which the cyano group is replaced by an electron-withdrawing ring system. Implementation of these altered NASA groups into the design of GPCR-targeting probes might therefore be a solution to increase the proportion of labeled target GPCR to off-target protein. For example, replacing the cyano moiety with an aryl group might increase the half-life time of potential NASA-containing ligands by over 50-fold.^42^ However, in such a follow-up study also other warheads should be explored, such as the acyl imidazole or 2-fluorophenylester.^22,39^

In conclusion, we have explored ligand-directed labeling of A_2B_AR, an exemplary GPCR that is an interesting target in cancer drug discovery. We have synthetically implemented the recently reported NASA warhead into previously developed ligands and show that the binding of A_2B_AR is not fully restricted by the acyl substitution. Furthermore, we have investigated ligand-directed labeling of the A_2B_AR using SDS-PAGE, flow cytometric, and mass spectrometry techniques. We have found that one of the synthesized ligand-directed probes labeled the A_2B_AR, however, also caused nonspecific labeling due to the combination of high intrinsic reactivity and low expression levels of the A_2B_AR. The herein synthesized probes show good binding toward the A_2B_AR, as well as labeling of the A_2B_AR in biochemical assays, but a significant reduction in A_2B_AR activation postlabeling and hampered detection when used in complex mixtures of proteins. In the future, targeting different amino acid residues on the A_2B_AR (e.g., distal from the binding pocket), and rationally tuning the NASA group might yield probes that are more biologically orthogonal and less prone to nonspecific protein labeling, as exemplified in the work by Hamachi et al.^42^ Ligand-directed probes bearing these properties will be valuable tools to study the A_2B_AR, and other GPCRs, upon ligand-induced activation.

## Methods

See the Supporting Information.

## Supporting Information Available

Additional table and figures, experimental procedures, and NMR spectra. Output tables pull-down experiments (PDF)
(XLSX)
(XLS)
