# Supplementary material for: N-Acyl-N-Alkyl Sulfonamide Probes for Ligand-Directed Covalent Labeling of GPCRs: The Adenosine A2B Receptor as Case Study
Source: ACS Chem Biol. 2024 Jun 26;19(7):1554–62. doi: 10.1021/acschembio.4c00210 (PMC11267576; doi:10.1021/acschembio.4c00210)
Supplement: Supplementary file 1 — cb4c00210_si_001.pdf [file cb4c00210_si_001.pdf]

Supporting Information

N-acyl-*N*-alkyl sulfonamide probes for ligand-directed covalent labeling of GPCRs: the adenosine A_{2B} receptor as case study

Bert L.H. Beerkens, Vasiliki Andrianopoulou, Xuesong Wang, Rongfang Liu, Gerard J.P. van Westen, Willem Jespers, Adriaan P. IJzerman, Laura H. Heitman and Daan van der Es

Table of Contents

Table of Contents 2
Supplemental Table 3
Supplemental Figures 4
Chemistry 6
  General Chemistry 6
  Synthesis of ligand-directed probes 6
Computational procedures 9
  Molecular docking 9
Biological procedures 10
  Materials 10
  Cell culture and membrane preparation 10
  Radioligand displacement assays 10
  Functional cAMP assay 11
  SDS-PAGE assays 11
    Labeling experiments in CHO-A_{2B}AR membrane fractions 11
    Labeling experiments in live CHO-A_{2B}AR cells 11
    Data Analysis and statistics 12
  Flow cytometry experiments 12
  Proteomic pull-down experiments 12
    Probe binding, click reaction, protein precipitation, reduction and alkylation 12
    Pull-Down, digestion and de-salting 13
    LC-MS settings 13
    Data analysis 14
NMR Spectra 15
References 20

Supplemental Table

Table S1. Radioligand displacement experiments on other adenosine receptors.

	Displacement at 1 µM (%)		
**compound**	**A_1AR[a]**	**A_{2A}AR[b]**	**A_3AR[c]**
**1**	29 (28, 30)	52 (59, 46)	7 (11, 3)
**3a**	19 (21, 17)	9 (6, 12)	-5 (-8, -2)
**3b**	82 (82, 81)	0 (-3, 3)	6 (-2, 14)
**4a**	34 (30, 38)	40 (35, 45)	-2 (-8, 5)
**4b**	46 (46, 46)	27 (15, 38)	-9 (-14, -3)

[a] % specific [^{3}H]DPCPX displacement by the respective probe on CHO cell membranes stably expressing the hA_1AR at 25 °C after 0.5 h of co-incubating probe and radioligand; [b] % specific [^{3}H]ZM241385 displacement by the respective probe on HEK293 cell membranes stably expressing the hA_{2A}AR at 25 °C after 0.5 h of co-incubating probe and radioligand; [c] % specific [^{3}H]PSB11 displacement by the respective probe on CHO cell membranes stably expressing hA_3AR at 25 °C after 0.5 h of co-incubating probe and radioligand. Data represent the mean of two individual experiments performed in duplicate.

Supplemental Figures

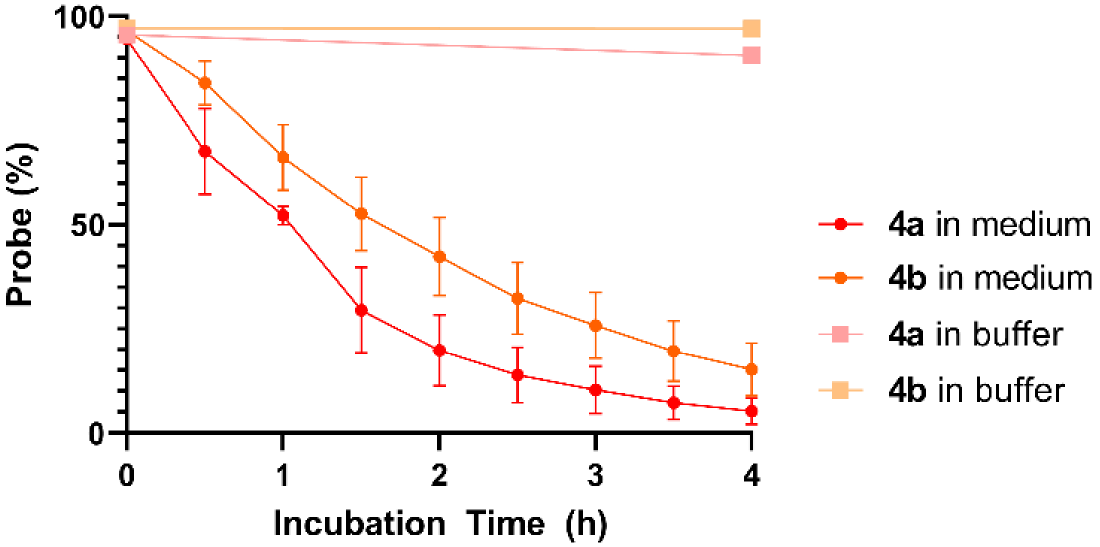

Figure S1. Investigation of probe stability and reactivity in buffer and medium. Probes **4a** and **4b** were added to PBS buffer or cellular medium (DMEM/F12 medium containing 10% (v/v) newborn calf serum). Samples were shaken to prevent probe precipitation and measured by LC-MS every 30 minutes. Data shown is the mean ± SEM of three replicates.

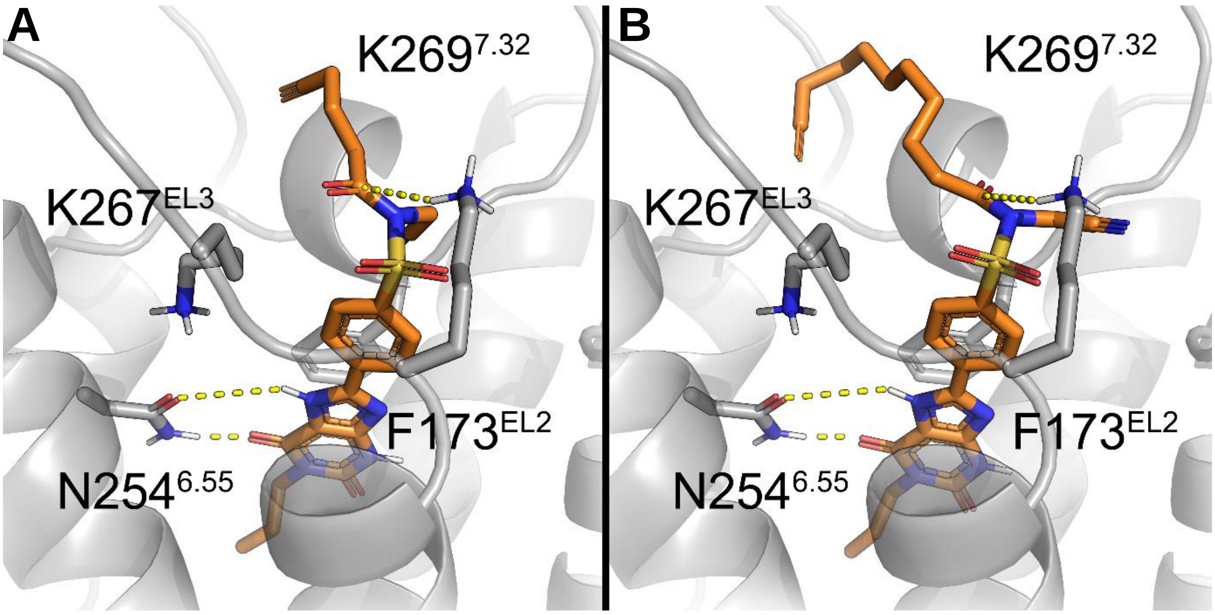

Figure S2. Predicted binding mode of probes **4a** (panel A) and **4b** (panel B) in the binding pocket of the $A_{2B}AR$. An antagonist-bound model of the $A_{2B}AR$ was used in which binding of the compounds, as well as the loop regions containing the presumed lysine nucleophiles, correspond to the recently published agonist-bound cryo-EM structure.[1] Key interacting amino acid residues are shown, including the amino acids known for binding xanthine antagonists in adenosine receptors, N254 and F173,[2] as well as the potential lysine nucleophiles K267 and K269.[3,4] Hydrogen bonds are depicted as yellow dashed lines. K269 is the nucleophilic amino acid most closely oriented towards the electrophilic carbonyl group of the NASA warhead and therefore likely is the 'main' nucleophile. Next to that, K267 also resides in close proximity to the NASA group and might therefore also act as nucleophile in some cases, for example when K269 is mutated.[4]

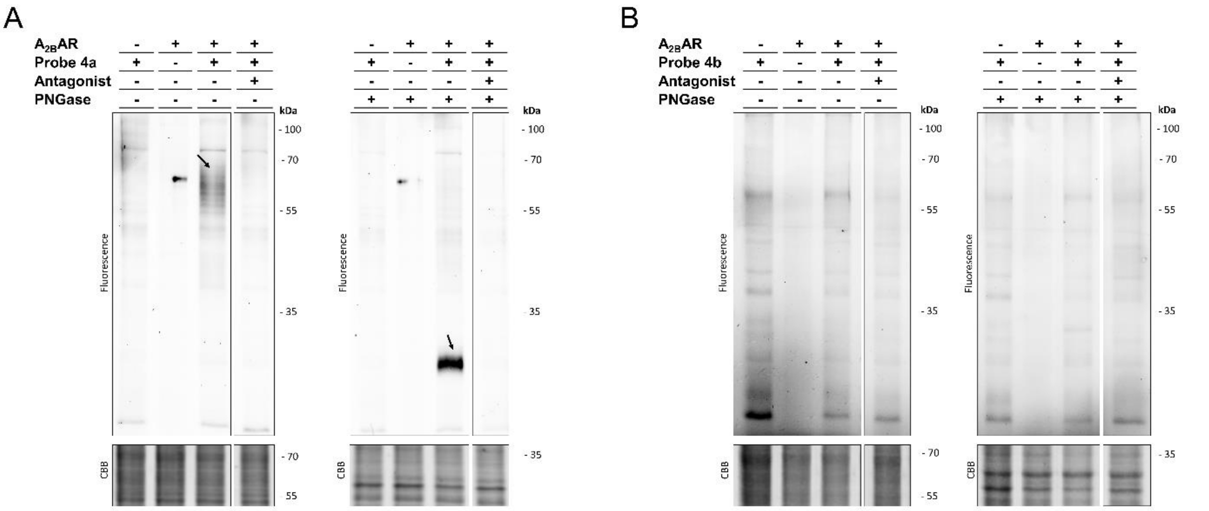

Figure S3. Ligand-directed labeling of the $A_{2B}AR$ in live CHO cells using an probe incubation time of 2 h instead of 30 min. All other conditions were the same as for the experiment in Figure 4. In summary, CHO cells with or without stable expression of the $A_{2B}AR$ were pre-incubated for 30 min with medium containing either 1% DMSO (vehicle) or 10 µM of irreversible antagonist **1**. The cells were subsequently incubated for 30 min with 400 nM probe in Hank's Balanced Salt Solution or 1% DMSO (vehicle control). Cells were washed with PBS and membranes were collected. N-glycans were removed using PNGase (5 U from a 10 U/µL solution) and alkyne moieties were clicked to 1 µM Cy5-N_3. The samples were then denatured using Laemmli buffer and resolved by SDS-PAGE. Gels were imaged by in-gel fluorescence. Coomassie Brilliant Blue (CBB) staining was used as loading control. (A) Protein labeling by **4a**. The arrows indicate the presumable band of the $A_{2B}AR$. (B) Protein labeling by **4b**. Gels are representatives of three replicates.

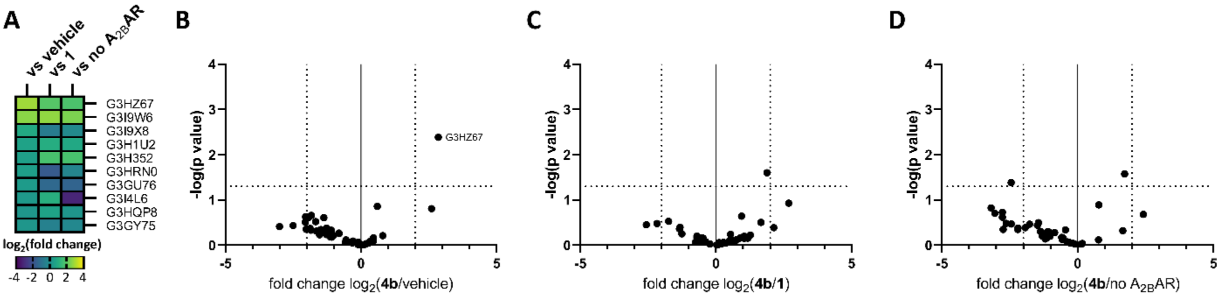

Figure S4. Proteomic pull-down experiments using probe **4b**. Shown are the $\log_2$(fold changes) depicting the intensity scores of probe-labeled proteins in positive samples, divided by the intensity scores of probe-labeled proteins in various control samples (vehicle, pre-incubation with antagonist **1** and without expression of the $A_{2B}AR$). Fold changes are depicted by color in case of the heat map (A) and on the x-axis of the volcano plots (B-D). Vasohibin-2 (G3HZ67) was found to be enriched as compared to the vehicle samples (B; upper right corner), however, none of the proteins was found to have a significant fold change upon pre-incubation with irreversible antagonist **1**. CHO cells with or without ('no $A_{2B}AR$') stable expression of the $A_{2B}AR$ were pre-incubated for 30 min with medium containing either 1% DMSO (vehicle) or 10 µM of irreversible antagonist **1** ('**1**'). The cells were subsequently incubated for 30 min with 400 nM probe in Hank's Balanced Salt Solution ('**4b**') or 1% DMSO (vehicle control; 'vehicle'). Cells were washed with PBS and membranes were collected. Alkyne moieties were clicked to Biotin-N_3, reduced, alkylated and pulled-down using avidin beads. Bound proteins were digested to peptides, desalted and analyzed by LC-MS/MS. (A) Heat map of the proteins labeled by probe **4b**. Shown are the top 10 of proteins that showed the highest fold change over vehicle. (B-D) Volcano plot comparing the **4b** labeled proteins towards various control conditions, such as vehicle, pre-incubation with antagonist **1** and CHO cells without expression of the $A_{2B}AR$. Fold change is depicted on the x-axis and -log(p value) on the y-axis. Protein IDs (CHO proteins) are given and taken from Uniprot. Data originates from three replicates.

Chemistry

General Chemistry

All reactions were performed using commercially available chemicals and solvents, purchased via Sigma-Aldrich (Merck), VWR chemicals and Thermo Scientific. All reactions were carried out under an N_2 atmosphere and at room temperature, unless noted otherwise. Thin layer chromatography (TLC) was carried out using TLC Silica Gel 60 F254 (Merck) and visualized using UV irradiation at wavelengths of 254 and 366 nM. ^{1}H, ^{13}C and ^{19}F NMR spectra were recorded on a Bruker AV-400 (400 MHz) or Bruker AV-500 (500 MHz) spectrometer. Chemical shift values are reported in parts per million (ppm) and designated by δ. Tetramethylsilane or solvent resonance was used as internal standard. Coupling constants (J) are reported in Hertz (Hz) and multiplicities are indicated by s (singlet), bs (broad singlet), d (doublet), t (triplet), td (triplet of doublets), p (pentuplet), h (hexuplet) or m (multiplet). Compound purity was determined by HPLC-MS, using a LCMS-2020 system coupled to a Gemini® 3 µm C18 110Å column (50 x 3 mm). Samples were dissolved in H_2O:MeCN:*t*-BuOH 1:1:1, injected onto the column and eluted with a gradient of H_2O:MeCN 9:1 + 1% formic acid to H_2O:MeCN 1:9 + 1% formic acid over the course of 15 minutes. High-resolution mass spectrometry (HRMS) measurements were done on a X500R QTOF mass spectrometer (SCIEX).

Synthesis of ligand-directed probes

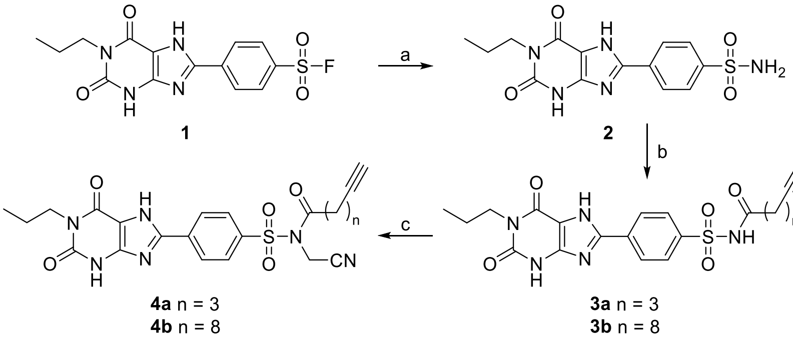

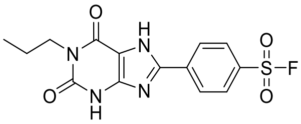

4-(2,6-Dioxo-1-propyl-2,3,6,7-tetrahydro-1*H*-purin-8-yl)benzenesulfonyl fluoride (1)
Compound **1** was synthesized as reported recently.[3]

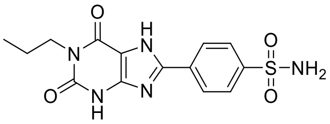

4-(2,6-Dioxo-1-propyl-2,3,6,7-tetrahydro-1*H*-purin-8-yl)benzenesulfonamide (2)
Sulfonyl fluoride **1** (1.6 g, 4.54 mmol, 1.0 eq) was dissolved in 28-30% ammonia solution (23 mL) and stirred for 2 h. The reaction mixture was then quenched and acidified by dropwise addition of 6 M HCl (60 mL). The product was crystallized overnight and the residue was collected by vacuum filtration. To remove impurities, the residue was dissolved in 0.5 M NaOH (75 mL) and the aqueous mixture was washed using 100 mL of 5% MeOH in $CHCl_3$. The pH was then brought to ~2 using 6 M HCl and the product was allowed to crystallize overnight. The residue was collected by vacuum filtration and dried under vacuum to yield **2** (1.19 g, 3.40 mmol, 75 %) as a purple solid, which was used in the next steps without further purification.

^{1}H NMR (400 MHz, $(CD_3)_2SO$) δ [ppm] = 11.78 (bs, 1H), 8.21 (d, J = 8.6 Hz, 2H), 7.87 (d, J = 8.3 Hz, 2H), 7.41 (s, 2H), 3.81 (t, J = 7.5 Hz, 2H), 1.56 (h, J = 7.6 Hz, 2H), 0.87 (t, J = 7.4 Hz, 3H).

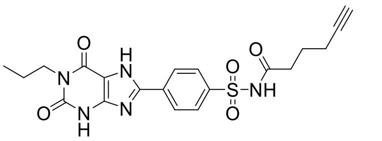

***N*-((4-(2,6-Dioxo-1-propyl-2,3,6,7-tetrahydro-1*H*-purin-8-yl)phenyl)sulfonyl)hex-5-ynamide (3a)**

5-hexynoic acid (98 µL, 0.89 mmol, 1.6 eq), EDC·HCl (316 mg, 1.65 mmol, 3.0 eq), DMAP (20 mg, 0.17 mmol, 0.3 eq) and DIPEA (290 µL, 1.66 mmol, 3.0 eq) were added to a solution of sulfonamide **5** (192 mg, 0.55 mmol,1.0 eq) in dry DMF (6 mL) and the mixture was stirred overnight. EtOAc (50 mL) was added and the organic layer was washed with H_2O (3 x 50 mL). As the product resided in the aqueous layer, the aqueous layers were combined and the pH was brought to ~2 using 6 M HCl. The product was allowed to crystallize overnight and collected by vacuum filtration. The filtrate was recrystallized and the second residue was collected by vacuum filtration. The residues were combined and purified using column chromatography (DCM:MeOH 98:2→93:7) to yield **6** (101 mg, 0.03 mmol, 41%) as an off-white solid.

^{1}H NMR (500 MHz, $(CD_3)_2SO$) δ [ppm] = 14.03 (s, 1H), 11.99 (s, 1H), 8.28 (d, J = 8.7 Hz, 2H), 8.00 (d, J = 8.7 Hz, 2H), 3.82 (t, J = 7.3 Hz, 2H), 2.76 (t, J = 2.6 Hz, 1H), 2.32 (t, J = 7.4 Hz, 2H), 2.08 (td, J = 7.1, 2.7 Hz, 2H), 1.61 – 1.53 (m, 4H), 0.88 (t, J = 7.4 Hz, 3H).

^{13}C NMR (126 MHz, DMSO) δ 172.2, 155.5, 151.5, 148.6, 148.1, 141.0, 133.7, 128.7, 127.2, 109.1, 84.2, 72.3, 42.0, 34.9, 23.4, 21.4, 17.5, 11.7.

HRMS (ESI, m/z): $[M+H]^+$, calculated: 444.1336, found: 444.1308.

HPLC 97%, RT 8.797 min.

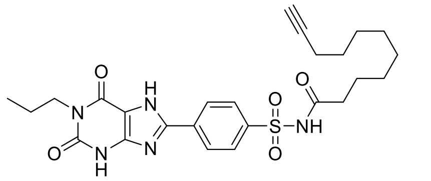

***N*-((4-(2,6-Dioxo-1-propyl-2,3,6,7-tetrahydro-1*H*-purin-8-yl)phenyl)sulfonyl)undec-10-ynamide (3b)**

10-Undecynoic acid (328 mg, 1.80 mmol,1.6 eq), EDC·HCl (656 mg, 3 mmol, 3 eq), DMAP (42 mg, 0.34 mmol, 0.3 eq) and DIPEA (600 µL, 3.44 mmol, 3.0 eq) were added to a solution of **5** (400 mg, 1.14 mmol, 1.0 eq) in dry DMF (12 mL) and the mixture was stirred overnight. EtOAC (100 mL) was then added and the organic layer was extracted with H_2O (3 x 100 mL). As the product resided in the aqueous layer, the aqueous layers were combined and the pH was brought to ~2 using 6 M HCl. The product was allowed to crystallize overnight and was collected by filtration. The product was further purified by recrystallization in a 1:4 mixture of H_2O:EtOAc to yield compound **8** as a pink-white solid (376 mg, 0.73 mmol, 64%).

^{1}H NMR (400 MHz, $(CD_3)_2SO$) δ [ppm] = 14.05 (s, 1H), 12.16 (s, 1H), 11.99 (s, 1H), 8.29 (d, J = 8.6 Hz, 2H), 8.00 (d, J = 8.6 Hz, 2H), 3.82 (t, J = 7.4 Hz, 2H), 2.69 (t, J = 2.6 Hz, 1H), 2.20 (t, J = 7.2 Hz, 2H), 2.06 (td, J = 7.0, 2.7 Hz, 2H), 1.57 (h, J = 7.5 Hz, 2H), 1.43 – 1.28 (m, 4H), 1.27 – 1.18 (m, 2H), 1.17 – 1.02 (m, 6H), 0.88 (t, J = 7.4 Hz, 3H).

^{13}C NMR (101 MHz, $(CD_3)_2SO$) δ [ppm] = 172.3, 155.4, 151.4, 148.4, 148.1, 140.5, 133.8, 128.7, 127.1, 109.0, 84.9, 71.5, 42.0, 35.8, 29.0, 28.8, 28.6, 28.5, 28.4, 24.4, 21.3, 18.1, 11.7.

HRMS (ESI, m/z): $[M+H]^+$, calculated: 514.2119, found: 514.2089.

HPLC 100%, RT 10.459 min.

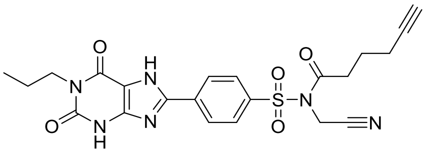

N-(Cyanomethyl)-N-((4-(2,6-dioxo-1-propyl-2,3,6,7-tetrahydro-1H-purin-8-yl)phenyl)sulfonyl)hex-5-ynamide (4a)

Bromoacetonitrile (8 µL, 0.11 mmol,1.2 eq) was added to a solution of **6** (43 mg, 0.10 mmol, 1.0 eq) in dry DMF (3 mL) and the mixture was stirred overnight. A small conversion of starting material was observed and therefore extra bromoacetonitrile (4 µL, 0.06 mmol, 0.05 eq) was added. The mixture was further stirred for 8 days during which DIPEA (14 µL, 0.08 mmol, 0.8 eq) was added gradually. The reaction was then stopped to prevent overalkylation. EtOAc (50 mL) was added and the organic layer was washed with H_2O (3 x 50 mL), dried using $MgSO_4$, filtered and concentrated under reduced pressure. The residue was purified using column chromatography (DCM: MeOH 99.5:0.5→ 97.5:2.5) to yield **7** (20 mg, 0.04 mmol, 41%) as white solid. Substitution of the cyano group at the sulfonamide position was confirmed by coupling between the cyanomethyl protons (CH_2) and the sulfonamide carbonyl group in Heteronuclear Multiple Bond Correlation (HMBC) measurements, as indicated by the inlet in the NMR spectrum below.

^{1}H NMR (500 MHz, $(CD_3)_2SO$) δ [ppm] = 14.10 (s, 1H), 12.01 (s, 1H), 8.35 (d, J = 8.6 Hz, 2H), 8.16 (d, J = 8.7 Hz, 2H), 4.98 (s, 2H), 3.82 (t, J = 7.4 Hz, 2H), 2.81 (t, J = 7.2 Hz, 2H), 2.77 (t, J = 2.6 Hz, 1H), 2.16 (td, J = 7.0, 2.7 Hz, 2H), 1.65 (p, J = 7.1 Hz, 2H), 1.57 (h, J = 7.4 Hz, 3H), 0.88 (t, J = 7.4 Hz, 3H).

^{13}C NMR (126 MHz, $(CD_3)_2SO$) δ [ppm] = 172.2, 155.5, 151.5, 148.1, 148.1, 138.7, 134.9, 129.1, 127.6, 116.9, 109.4, 84.0, 72.5, 42.1, 34.7, 34.5, 23.4, 21.4, 17.3, 11.7.

HRMS (ESI, m/z): $[M+H]^+$, calculated: 483.1445, found: 483.1423.

HPLC 96%, RT 9.721 min.

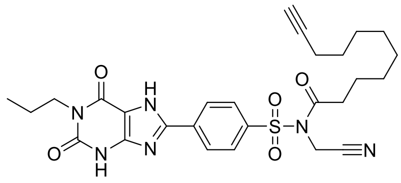

N-(Cyanomethyl)-N-((4-(2,6-dioxo-1-propyl-2,3,6,7-tetrahydro-1H-purin-8-yl)phenyl)sulfonyl)undec-10-ynamide (4b)

Bromoacetonitrile (21 µL, 0.30 mmol, 1.2 eq) was added to a solution of **8** (130 mg, 0.25 mmol, 1.0 eq) in dry DMF (8 mL). DIPEA (14 µL, 0.08 mmol, 0.3 eq) was added gradually over a period of six days. The reaction was then stopped to prevent overalkylation. EtOAc (100 mL) was added and the organic layer was washed using H_2O (3 x 100mL), dried over $MgSO_4$ and concentrated under reduced pressure. The residue was purified using column chromatography (DCM: MeOH 99.5:0.5→98:2) to yield **9** (16 mg, 0.02 mmol, 12%) as a white solid. Substitution of the cyano group at the sulfonamide position was confirmed by coupling between the cyanomethyl protons (CH_2) and the sulfonamide carbonyl group in HMBC measurements, as indicated by the inlet in the NMR spectrum below.

^{1}H NMR (500 MHz, $(CD_3)_2SO$) δ [ppm] = 14.11 (s, 1H), 12.00 (s, 1H), 8.35 (d, J = 8.8 Hz, 2H), 8.15 (d, J = 8.7 Hz, 2H), 5.00 (s, 2H), 3.82 (t, J = 7.3 Hz, 2H), 2.67 (t, J = 2.7 Hz, 1H), 2.64 (t, J = 7.2 Hz, 2H), 2.06 (td, J = 7.0, 2.7 Hz, 2H), 1.58 (h, J = 7.4 Hz, 2H), 1.47 – 1.40 (m, 2H), 1.38 – 1.29 (m, 2H), 1.25 – 1.20 (m, 2H), 1.17 – 1.09 (m, 6H), 0.88 (t, J = 7.5 Hz, 3H).

^{13}C NMR (126 MHz, $(CD_3)_2SO$) δ [ppm] = 172.7, 155.5, 151.5, 148.1, 148.0, 139.0, 134.8, 129.1, 127.5, 117.0, 109.3, 85.0, 71.5, 42.1, 35.6, 34.5, 29.0, 28.8, 28.6, 28.5, 28.4, 24.4, 21.4, 18.1, 11.7.

HRMS (ESI, m/z): $[M+H]^+$, calculated: 553.2228, found: 553.2218.

HPLC 98%, RT 11.300 min.

Computational procedures

Molecular docking

A previously published apo structure model of adenosine A_{2B} receptor was used as a starting point for docking.[3] The protein was prepared for molecular docking using Maestro's (v2022-3) protein preparation wizard, including an energy minimization step. Thereafter, a docking grid was generated around the binding site and prepared for docking with GLIDE.[5] Compounds **4a** and **4b** were generated using LigPrep,[6] and consequently docked using GLIDE. The highest scoring docking poses were retained. Images were generated using PyMOL version 2.5.2.[7]

Biological procedures

Materials

Radioligands: [^{3}H]PSB-603 (specific activity 79 Ci/mmol) was purchased from Quotient Bioresearch, [^{3}H]DPCPX, (specific activity 137 Ci/mmol) and [^{3}H]-ZM241385 (specific activity 50 Ci/mmol) were purchased from ARC, Inc. and [^{3}H]PSB-11 (specific activity 56 Ci/mmol) was kindly donated by Prof. C.E. Müller (University of Bonn, Germany). **Small molecules:** 5'-(N-Ethylcarboxamido)adenosine (NECA), N^6-Cyclopentyladenosine (CPA), and EDTA-free protease inhibitor cocktail (cat# P8340) and biotin-PEG3-azide were purchased from Sigma-Aldrich (Merck). ZM241385 was a gift of Dr. S.M. Poucher (Astra Zeneca, Manchester, UK) and CGS21680 was purchased from Ascent Scientific. PSB 1115 potassium salt was purchased from Tocris Bioscience. Cilostamide was ordered from Santa Cruz Biotechnology. Forskolin was ordered from Toronto Research Chemicals Inc. and rolipram was ordered from Cayman Chemical Company. **Click reagents:** All click reagents, i.e. $CuSO_4$, (+)-sodium L-ascorbate (NaAsc), Tris((1-hydroxy-propyl-1H-1,2,3-triazol-4-yl)methyl)amine (THPTA), Cyanine5-N_3 (Cy5-N_3) and biotin-N_3 were purchased from Merck. **Proteins:** Adenosine deaminase (ADA) was purchased from Merck. PNGase F (10 u/µL, cat# V4831) and Chymotrypsin (cat# V1061) were purchased from Promega and Bovine Serum Albumin (BSA) and Pierce™ Avidin agarose beads (cat # 11846734) were ordered from Thermo Scientific. **Buffers:** Hank Buffered Saline Solution (HBSS) was ordered from Thermo Scientific. Laemmli buffer was purchased from Bio-Rad. All other reagents were purchased from standard commercial sources and of analytical grade.

Cell culture and membrane preparation

Chinese Hamster Ovary (CHO)-spap cells stably expressing the human adenosine A_{2B} receptor (CHO-A_{2B}AR) were kindly provided by S.J. Dowell (GlaxoSmithKline, Stevenage, UK), CHO cells stably expressing the human adenosine A_1 receptor (CHO-hA_1AR) were kindly provided by Prof. S.J. Hill (University of Nottingham, UK), Human Embryonic Kidney (HEK) 293 cells stably expressing the human adenosine A_{2A} receptor (HEK293-hA_{2A}AR) were kindly provided by Dr. J. Wang (Biogen, Cambridge, Massachusetts USA) and CHO cells stably expressing the human adenosine A_3 receptor (CHO-hA_3AR) were kindly provided by Dr. K.N. Klotz (University of Würzburg, Germany). All cells were cultured and membranes were prepared as reported before.[8]

Radioligand displacement assays

Radioligand displacement experiments with multiple concentrations of competitor were carried out using CHO-A_{2B}AR membranes, while single point displacement assays were carried out using CHO-hA_1AR, HEK293-hA_{2A}AR and CHO-hA_3AR membranes as previously reported.[3] Data was analyzed using GraphPad Prism 9.0 (Graphpad Software Inc., San Diego, California USA). IC_{50} values were obtained by non-linear regression curve fitting and converted to pIC_{50} values using the Cheng-Prusoff equation.[9] The K_D values of 1.7 nM of [^{3}H]PSB-603 at CHO-A_{2B}AR membranes and 1.6 nM of [^{3}H]DPCPX at CHO-hA_1AR membranes were taken from previous experiments.[10,11] The K_D values of 1.0 nM of [^{3}H]ZM241385 at HEK293-hA_{2A}AR membranes and 17.3 nM of [^{3}H]PSB11 at CHO-hA_3AR membranes were taken from in-house determinations. All pK_i values shown are mean values ± SEM of three individual experiments performed in duplicate. Single point displacement values are the mean percentages of two experiments performed in duplicate. Statistical analysis was performed using a two-tailed unpaired student's T-test.

Functional cAMP assay

$A_{2B}AR$ activation was measured using the cAMP LANCE Ultra Kit (PerkinElmer). In brief, CHO-$A_{2B}AR$ cells were pre-incubated for 30 min either with ('pre-incubation and wash-out') or without ('co-incubation') a single concentration (10x K_i) of the $A_{2B}AR$ ligands in assay medium (containing 0.8 IU/mL ADA; 0.1% DMSO). Cells were washed three times with assay medium, washed once with PBS, detached using PBS/EDTA, transferred to a 15 mL centrifuge tube, centrifuged (3 min, 1000 rpm) and resuspended in sample buffer (HBSS with 5 mM HEPES; 0.1% (w/v) BSA; 50 µM cilostamide; 50 µM rolipram, 50 µM and 0.8 IU/mL ADA). The cellular suspension was brought to 600.000 cells/mL and 5 µL was added to the wells of a 384 well plate. Next, the full agonist NECA (100 nM; approximate EC_{80} concentration) or 0.1% DMSO (vehicle control) in 5 µL assay medium was added and cells were incubated for 30 min at rt. Detection mix was freshly prepared by addition of the Eu-cAMP tracer (PerkinElmer) to the cAMP detection buffer (PerkinElmer) in a ratio of 1:50. 5 µL of the detection mix was added per well, followed by the addition of 5 µL of antibody solution (Ulight anti-cAMP (PerkinElmer):cAMP detection buffer 1:150). The plate was incubated for at least 1 h in the dark and measured by an EnVision Multilabel Reader (PerkinElmer). Data was analyzed by GraphPad Prism (version 9.0.0) and normalized according to the maximal response produced by the EC_{80} concentration of NECA. Given percentages are the mean values ± SEM of three experiments.

SDS-PAGE assays

Labeling experiments in CHO-$A_{2B}AR$ membrane fractions

CHO-$A_{2B}AR$ membrane fractions were collected as previously reported.[8] Protease inhibitor cocktail (1:100) was added, the membrane fractions were diluted to a concentration of 1 mg/mL and 19 µL was taken per sample. 1 µL of probe **3a**, **3b**, **4a** or **4b** (final concentration: 10, 100 or 1000 nM) was added and the samples were incubated for 2 h at rt. Click mix was freshly prepared by combining 5 parts 100 mM $CuSO_4$, 3 parts 1 M NaAsc, 1 part 100 mM THPTA and 1 part 100 µM Cy5-N_3. 2.22 µL of the prepared click mix was added per sample and the samples were incubated for 1 h at rt. Proteins were denatured by addition of 7.41 µL of Laemmli buffer (x4) and incubation for at least 1 h at rt. The samples were then loaded on gel (12.5% acrylamide) and run (180 V, 100 min). Gels were imaged on a Bio-Rad Universal Hood III using in-gel fluorescence. Pageruler prestained protein ladder was used as molecular weight marker. Coomassie Brilliant Blue (CBB) staining was carried out as control.

Labeling experiments in live CHO-$A_{2B}AR$ cells

CHO-$A_{2B}AR$ cells were cultured as previously reported.[8] Upon reaching ~90% confluency, medium was removed and competing ligand **1** (LUF7982) (final concentration: 10 µM) or 1% DMSO (vehicle) in medium was added, followed by a 30 min incubation (37 °C, 5% CO_2). Medium was then removed and probe **3a** or **3b** (final concentration: 400 nM) or 1% DMSO (vehicle) in HBSS was added, followed by a 30 min incubation (37 °C, 5% CO_2). HBSS was removed, non-bound probe was washed away with PBS and membranes were prepared as previously reported.[8] Protease inhibitor cocktail (1:100) was added, the membrane fractions were diluted to a concentration of 1 mg/mL and 20 µL was taken per sample. Click mix was freshly prepared by combining 5 parts 100 mM $CuSO_4$, 3 parts 1 M NaAsc, 1 part 100 mM THPTA and 1 part 100 µM Cy5-N_3. 2.22 µL of click mix was added per sample and the samples were incubated for 1 h at rt. 0.5 µL PNGase (10U/µL) was added and the samples were deglycosylated for 1 h at rt. The samples were denatured by addition of 7.57 µL Laemmli buffer (x4) and incubating for at least 1 h at rt. Samples were then loaded on gel (12.5% acrylamide) and run (180 V, 100 min). Gels were imaged using a Bio-Rad Universal Hood III and in-gel fluorescence. CBB staining was carried out as protein control.

Data Analysis and statistics

Gels were analyzed with ImageLab software version 6.0.1 (Bio-Rad). The adjusted volumes of the lanes were determined using the 'Lane Profile' tab and corrected for the amount of protein per lane, using the adjusted total lane volumes of the CBB stained gels. The lane intensity of 1000 nM of probe **4b** was set to 100% and the other lanes were normalized accordingly. Graphpad Prism (version 9.0.0) was used to calculate the mean values ± SEM of three individually performed experiments.

Flow cytometry experiments

CHO-A_{2B}AR cells were cultured as previously reported.[8] Cells were detached using PBS/EDTA and centrifuged (5 min, 1000 rpm). The pellet was dissolved in 1 mL of medium and brought to $2x10^6$ cells/mL. 100 µL of the cell suspension was added to the wells of a 96 well plate and the plate was centrifuged (5 min, 1000 rpm). Medium was replaced by medium containing 10 µM of competing ligand **1** (in 1% DMSO), the cells were resuspended and the plate was incubated for 30 min (37 °C, 5% CO_2). The plate was centrifuged (5 min, 1000 rpm) and medium was replaced by HBSS containing various concentrations of probe **4a** or **4b** (in 1% DMSO). Cells were resuspended and incubated for 30 min (37 °C, 5% CO_2), unless indicated otherwise. The plate was centrifuged (5 min, 1000 rpm), cells were washed with PBS and afterwards fixed for 15 min using a 4% paraformaldehyde in 10% formalin solution. The fixative was removed by washing with PBS and 20 mM glycine in PBS and the cells were permeabilized for 10 min using a solution of 0.1% saponin in PBS. The remaining saponin was removed by washing with PBS and the well plate was stored at 4 °C until further usage. At the day of measurements, PBS was removed and freshly prepared click mix was added containing, 1 part 100 mM $CuSO_4$, 1 part 1 M NaAsc, 1 part 100 mM THPTA, 997 parts PBS and 0.25 parts 40 µM Cy5-N_3. The plate was incubated with click reagents at rt for 1 h. Click mix was removed by washing with PBS and by incubation for 30 min with 1% BSA in PBS. Cells were measured on their mean fluorescence intensity (MFI) using a Cytoflex S (Beckman and Coulter, USA) and data was analyzed with FlowJo v10.0.7 (BD Life Sciences) and Graphpad Prism (version 9.0.0). Baseline-correction was performed by subtraction of the MFI values by the average MFI value of the vehicle-treated samples.

Proteomic pull-down experiments

Probe binding, click reaction, protein precipitation, reduction and alkylation

Pull-down experiments were carried out based on a recent protocol by van Rooden *et al.*[12] CHO-A_{2B}AR cells were cultured. Medium containing **1** (final concentration: 10 µM) or 1% DMSO (control) was added and the cells were incubated for 30 min (37 °C, 5% CO_2). Medium was removed and HBSS containing **4a** or **4b** (final concentration: 400 nM) or 1% DMSO (vehicle control) was added. The cells were incubated for 30 min (37 °C, 5% CO_2), after which non-bound probe was removed by washing with PBS. The cells were scraped, collected and membranes were prepared as previously reported.[8] Membrane fractions were diluted to a protein concentration of 2 mg/mL and 250 µL of proteins was taken per sample. Click mix was freshly prepared by adding 5 parts 100 mM $CuSO_4$, 3 parts 1 M NaAsc, 1 part 100 mM THPTA and 1 part 100 µM biotin-PEG3-N_3. 27.5 µL of click mix was added per sample and the samples were incubated for 1 h at rt. 92.5 µL of 10% SDS was added and the proteins were denatured for 1 h at rt. Protein precipitation was performed based on the method of Wessel and Flügge.[13] In brief, 800 µL MeOH, 400 µL $CHCl_3$ and 400 µL H_2O were added. The samples were vortexed and proteins were pelleted by centrifugation (10 min, 1500 G). The upper (aqueous) layers was removed, 600 µL of MeOH was added and proteins were pelleted by centrifugation (10 min, 1500 G). The supernatant was removed and proteins were resuspended in 500 µL 1% SDS containing 25 mM NH_4HCO_3. Probe sonication (Branson Sonifier 250, 3 x 5 s, 15%

amplitude) was necessary to fully dissolve the proteins. 10 µL of 0.5 M dithiothreitol (DTT) was added to reduce the proteins (15 min, 65 °C), followed by the addition of 80 µL of 0.25 M iodoacetamide to alkylate the free thiols (30 min, rt, dark). Another 10 µL of 0.5 M DTT was then added to quench the samples (15 min, rt).

Pull-Down, digestion and de-salting

Avidin agarose beads were taken (1400 µL for 12 samples) and divided over two 15 mL centrifuge tubes. The beads were washed three times by addition of PBS (4 mL), centrifugation (2 min, 2500 G) and removal of the supernatant. Washed beads were resuspended in 1.5 mL PBS per tube. In the meantime, protein samples were added to 15 mL tubes containing 9.1 mL PBS. 250 µL of bead solution was added to the protein samples (final SDS concentration: 0.05%) and the tubes were rotated (overnight, 4 °C). The next day, the beads were pelleted (2 min, 2500 G) and the supernatant was removed, The beads were brought to 1.5 mL Eppendorf tubes and washed with 1 mL 0.1% SDS in PBS, 1 mL PBS (3x) and 1 mL OB-DIG buffer (100 mM Tris-HCl pH 8.0, 100 mM NaCl, 10 mM $CaCl_2$ and 2% (v/v) acetonitrile),[12] by centrifugation (2 min, 2500 G) and removal of the supernatant. The beads were resuspended in 250 µL OB-DIG buffer, 2 µL chymotrypsin (1 µg) in 1 mM HCl was added and the proteins were digested (overnight, 37 °C, 1000 rpm). Resulting peptide solutions were desalted according to the Stage-Tip method.[14] In brief, Stage-Tips were pre-conditioned by 50 µL MeOH, 50 µL Solution B (0.5% formic acid in H_2O:MeCN 2:8) and 50 µL Solution A (0.5% formic acid in H_2O). Peptide samples were loaded on the Stage-Tips and washed with 100 µL of Solution A. The Stage-Tips were then brought to low-binding Eppendorf tubes and peptides were eluted by addition of 100 µL of Solution B. Solvents were evaporated using an Eppendorf concentrator. Samples were stored at -20 °C until further usage. At the day of measuring, peptides were reconstituted in 30 µL 0.1% formic acid in H_2O:MeCN 97:3 containing 10 fmol/µL yeast enolase digest (control).

LC-MS settings

Desalted peptides were separated on a Thermo Scientific Vanquish^{TM} Neo system using a Double nanoViper^{TM} PepMap^{TM} Neo 2 µm C18 75 µm x 150 mm column (Thermo Scientific). Peptides were separated with a 85 min LC method using mobile phase A (0.1% formic acid in ULC-MS grade water (Biosolve)) and mobile phase B (0.1% formic acid in 80% MeCN in ULC-MS grade water) controlled by a flow sensor at 0.3 µL/min with an average pressure of 300-400 bar. The method was programmed as a gradient with linear increment to 1% B from t=0 min to t=2 min, to 5% B at t=5 min, to 28% B at t=55 min, to 50% B at t=64 min, to 100% B at t=65 min and elution at 1% B between t=75 and t=85. The eluent was introduced by electro-spray ionization (ESI) via the nanoESI source (Thermo Scientific) using stainless steel Nano-bore emitters (cat# ES542, Thermo Scientific).

Eluted peptides were measured by an Orbitrap Exploris 240. The Orbitrap was operated in positive mode with data dependent acquisition (dda), without the use of lock mass, default charge of 2+ and external calibration with Pierce™ FlexMix™ Calibration Solution (cat# A39239, Thermo Scientific). The spray voltage was kept at 1.9 kV and the capillary temperature at 280 °C. The full scan was set to a resolution of 60000, scan range of 350-1400 m/z, RF-lens value of 80%, automatic gain control (AGC) of 1e6, max injection time of 50 ms, 1 microscan. For the data-dependent MS/MS events, the number of dependent scans was set to 15 and the general settings were isolation window of 1.6 m/z, higher-energy collisional dissociation (HCD) collision energy of 28%, resolution of 15,000, AGC target of 1e4, max injection time (IT) of 100 ms and 1 microscan. Unassigned +1 and charges>7 were excluded and the dynamic exclusion mode was set to 20 s after n=1 times.

Data analysis

Raw files were analyzed using MaxQuant (version 2.4.2.0).[15] A custom made fasta file was made for protein identification, containing the CHO proteome (Uniprot ID (UPID): UP000001075, downloaded January 12, 2021), the human $A_{2B}AR$ (P29275) and several background proteins, i.e. bovine serum albumin (P02769), chicken avidin (P02701), yeast enolase (P00924), bovine chymotrypsinogen (P00766) and streptavidin (P22629). Min. peptide length was set to 7 and max. peptide mass was set to 4600 Da. Oxidation (M) and Acetyl (Protein N-term) were set as variable modifications and Carbamidomethyl (C) as fixed modification, with a max. number of 5 modifications per peptide. Label-free quantification (LFQ) was turned on with a LFQ min. ratio count of 1 and fast LFQ enabled. Chymotrypsin+ was selected as digestion enzyme using a semi-specific digestion mode. The minimal amount of peptide for protein detection was set to 1 and match between runs was enabled. All other parameters were set according to the standard settings of MaxQuant.

The proteingroups.txt output tables were further analyzed using Perseus (version 2.0.11).[16] Proteins were removed that were only identified by site, reverse hits, potential contaminants and the background proteins mentioned above. Proteins without three valid LFQ values (>0) in at least one of the conditions were removed (potential false positives). The LFQ intensities of the remaining proteins were $\log_2(x)$ transformed and missing values were replaced by values from a normal distribution (width 0.3 and down shift 1.8). Samples were grouped according to their condition: +/- probe, +/- competing antagonist and +/- $A_{2B}AR$. Volcano plots were made with the volcano plot function, using a false-discovery rate of 0.05. Calculated x- and y-axis values were transferred to GraphPad Prism (version 9.0.0) for the final volcano plots and heat maps.

NMR Spectra

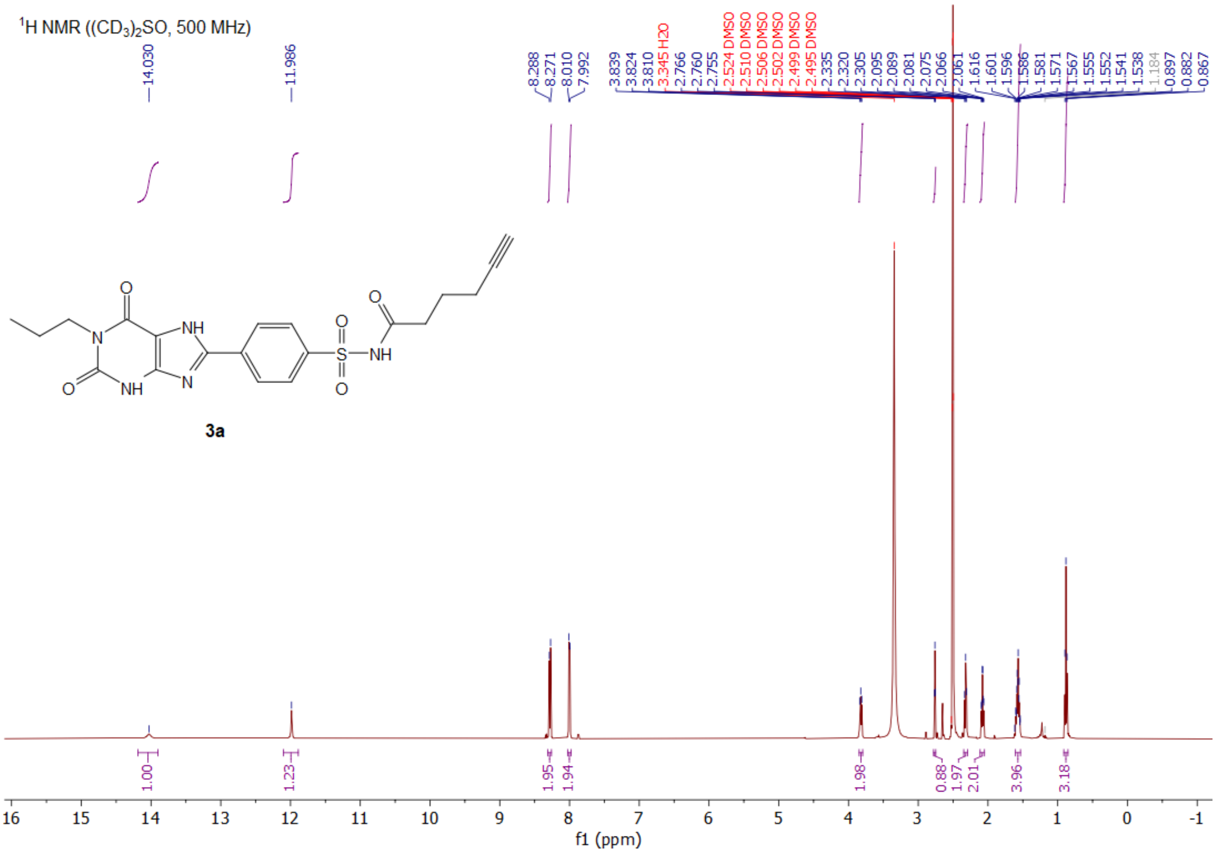

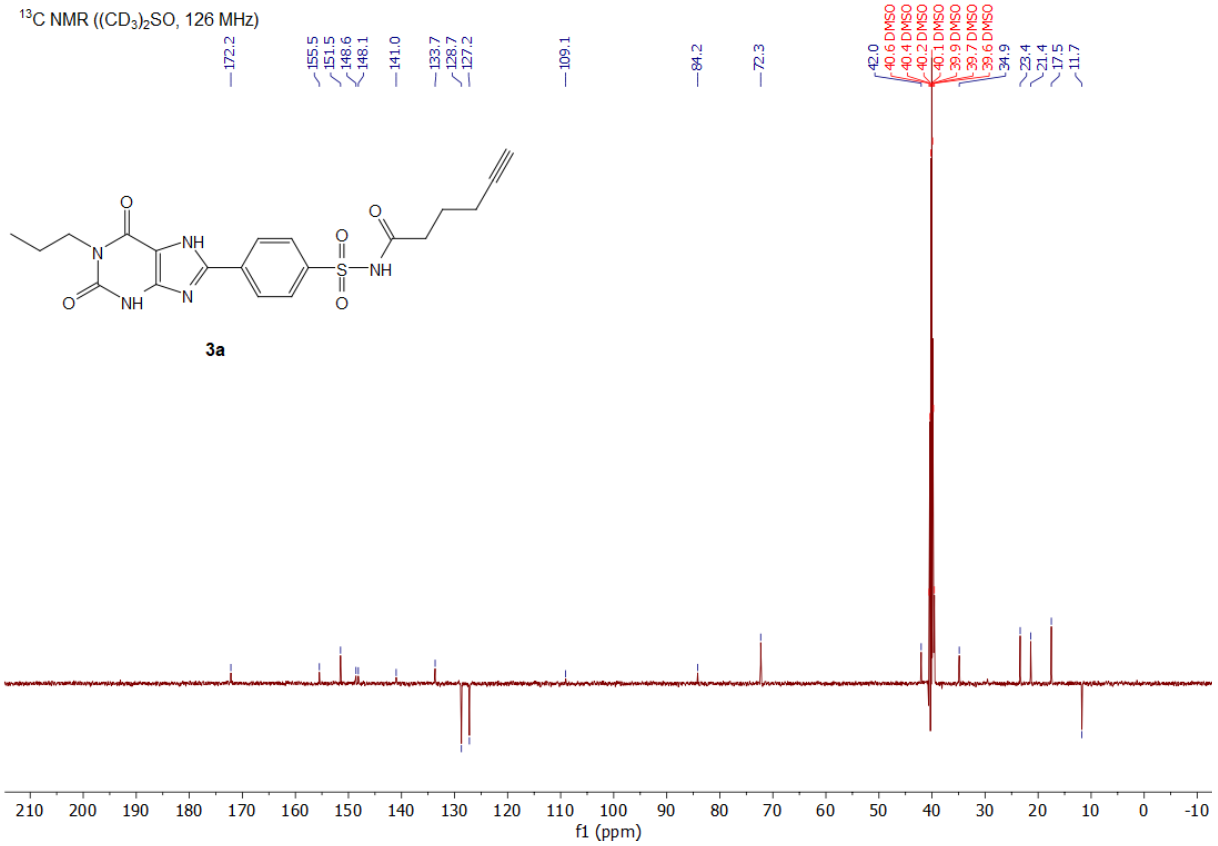

^{1}H NMR ($(CD_3)_2SO$, 400 MHz)

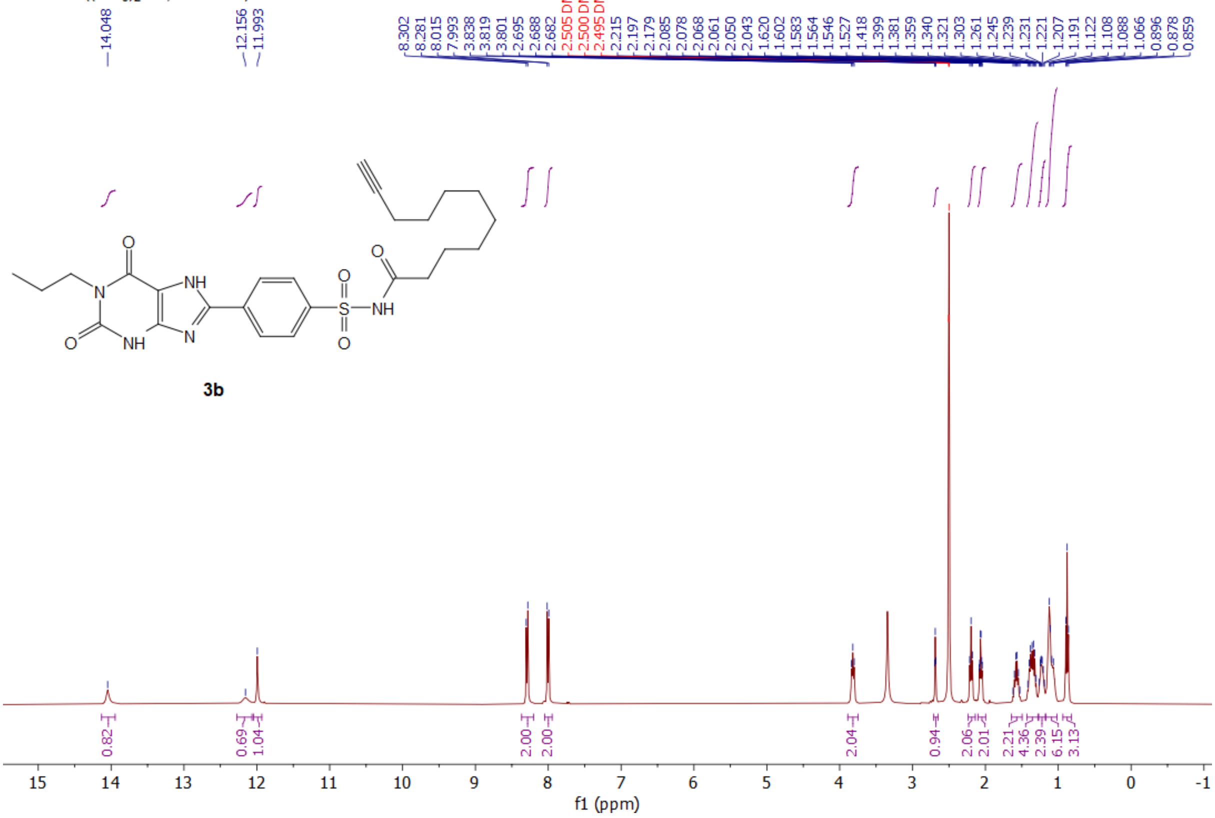

^{13}C NMR ($(CD_3)_2SO$, 101 MHz)

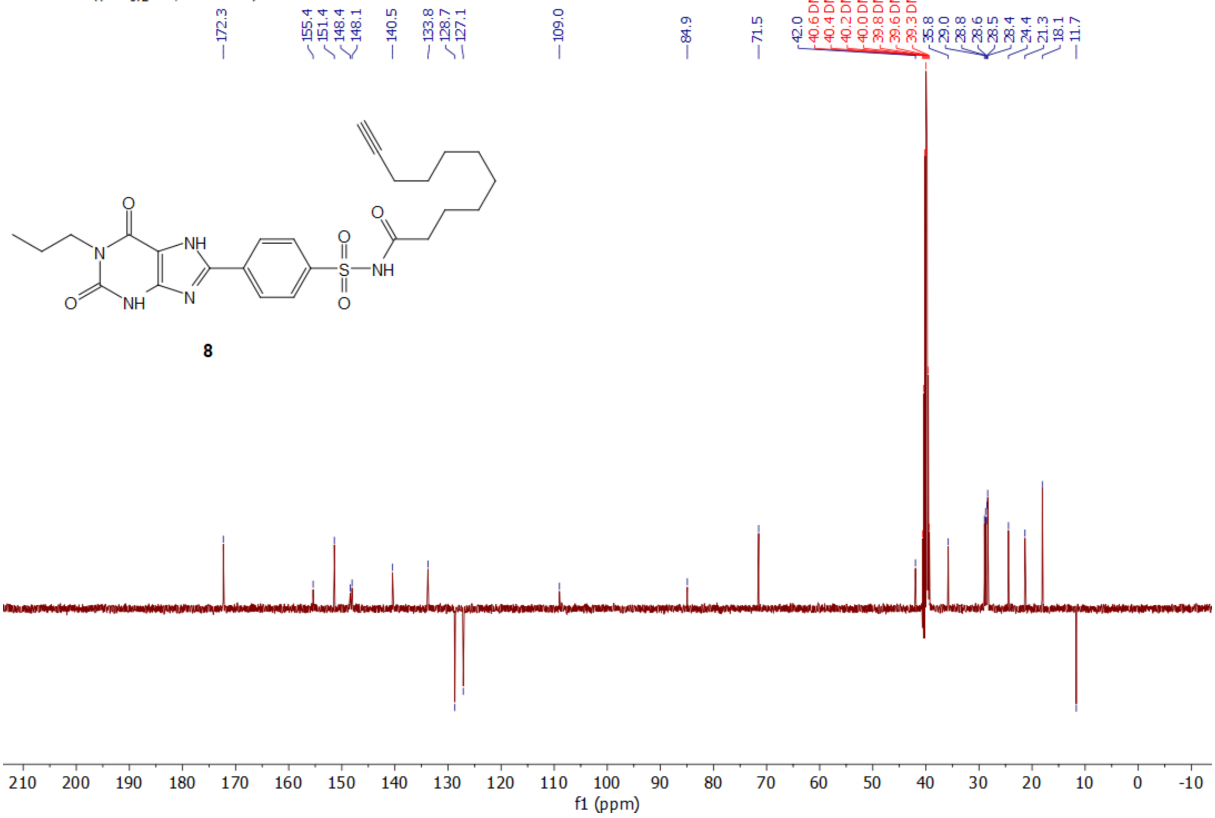

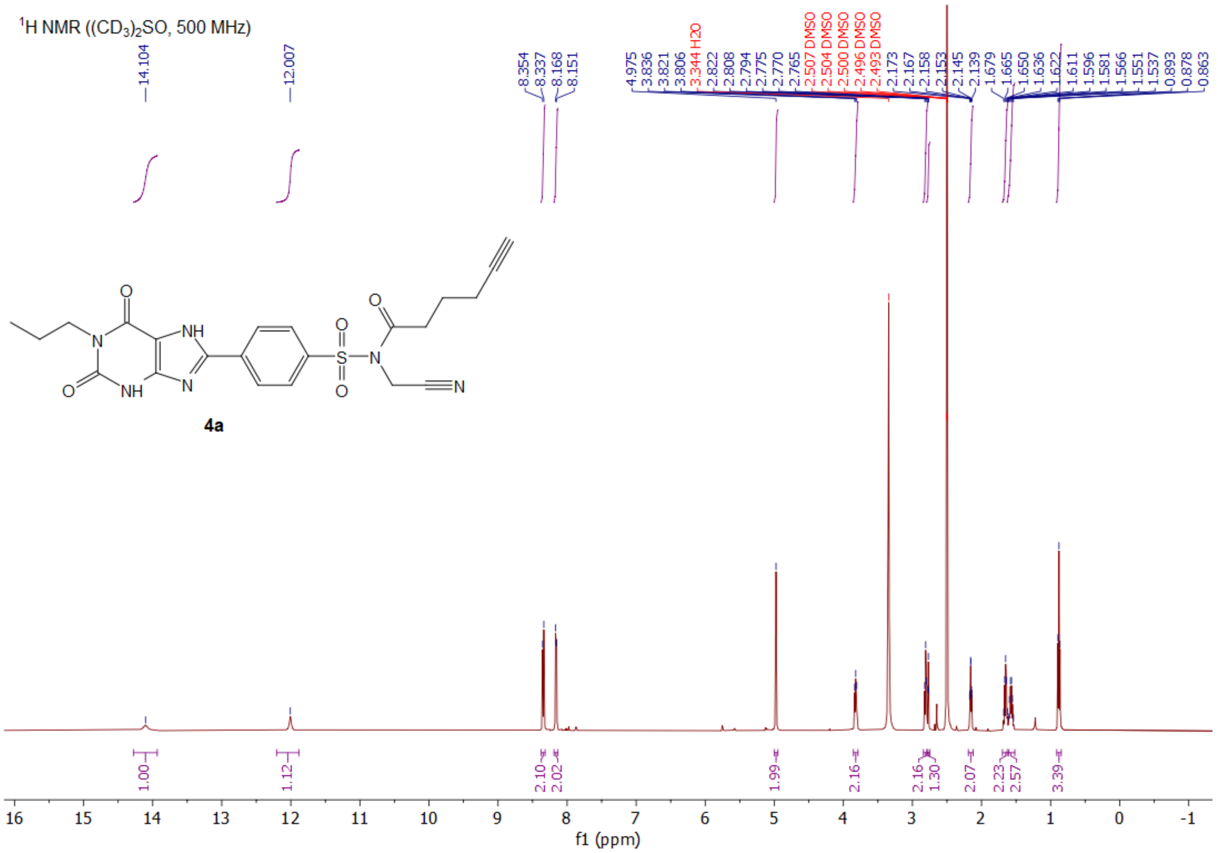
1H NMR ((CD3)2SO, 500 MHz)
14.104
12.007
8.354
8.337
8.168
8.151
4.975
3.836
3.821
3.806
3.344 H2O
2.822
2.808
2.794
2.775
2.770
2.765
2.507 DMSO
2.504 DMSO
2.500 DMSO
2.496 DMSO
2.493 DMSO
2.173
2.167
2.158
2.153
2.145
2.139
1.679
1.665
1.650
1.636
1.622
1.611
1.596
1.581
1.566
1.551
1.537
0.893
0.878
0.863
4a
1.00
1.12
2.10
2.02
1.99
2.16
2.16
1.30
2.07
2.23
2.57
3.39
f1 (ppm)

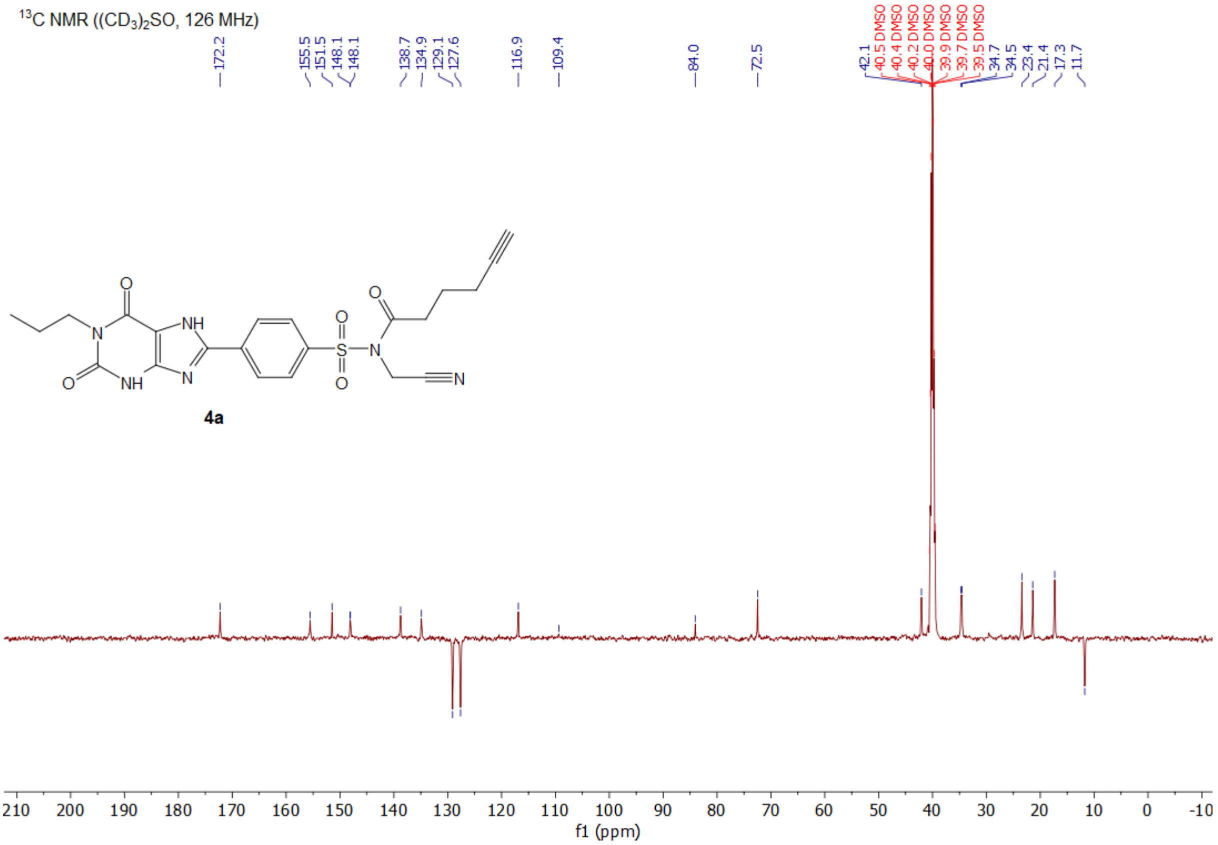
13C NMR ((CD3)2SO, 126 MHz)
172.2
155.5
151.5
148.1
148.1
138.7
134.9
129.1
127.6
116.9
109.4
84.0
72.5
42.1
40.5 DMSO
40.4 DMSO
40.2 DMSO
40.0 DMSO
39.9 DMSO
39.7 DMSO
39.5 DMSO
34.7
34.5
23.4
21.4
17.3
11.7
4a
f1 (ppm)

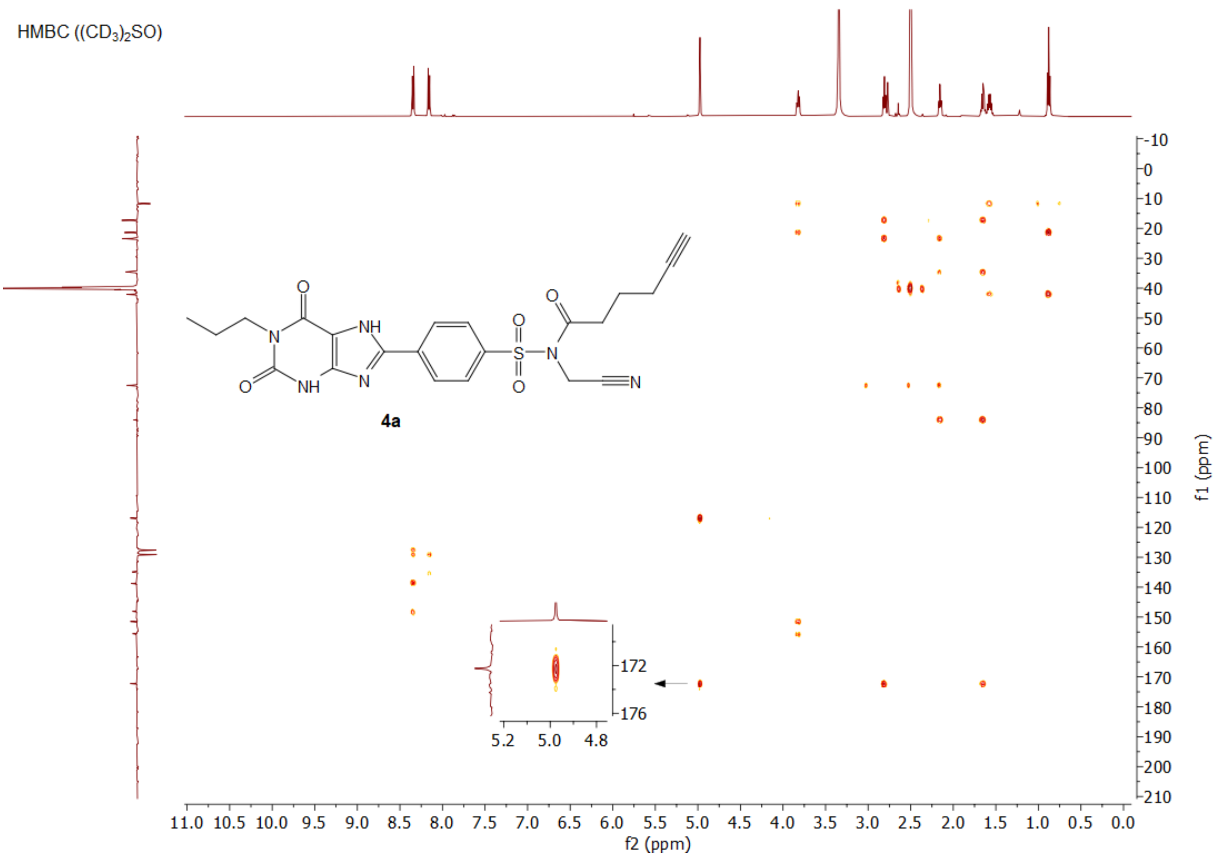
HMBC ((CD3)2SO)
4a
172
176
5.2
5.0
4.8
f2 (ppm)
f1 (ppm)

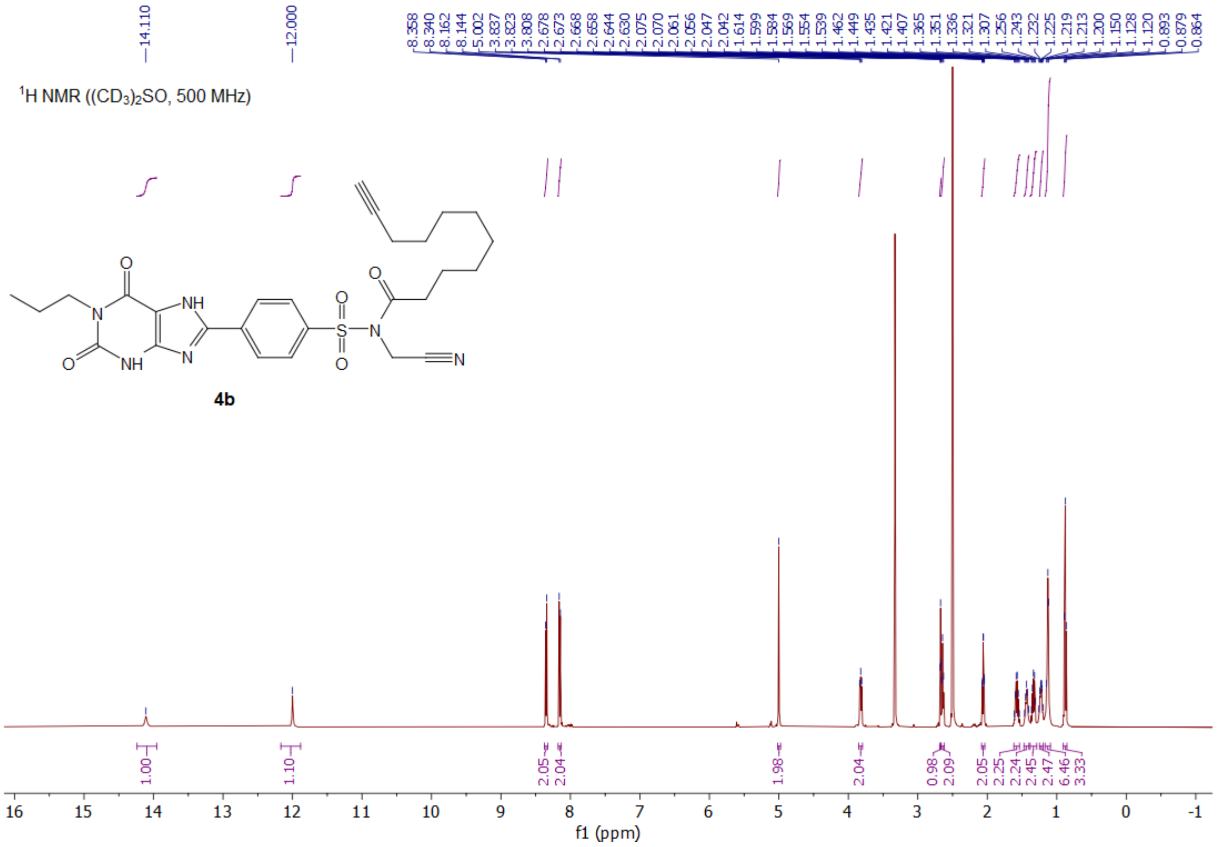
1H NMR ((CD3)2SO, 500 MHz)
4b
f1 (ppm)

^{13}C NMR $((CD_3)_2SO$, 126 MHz)

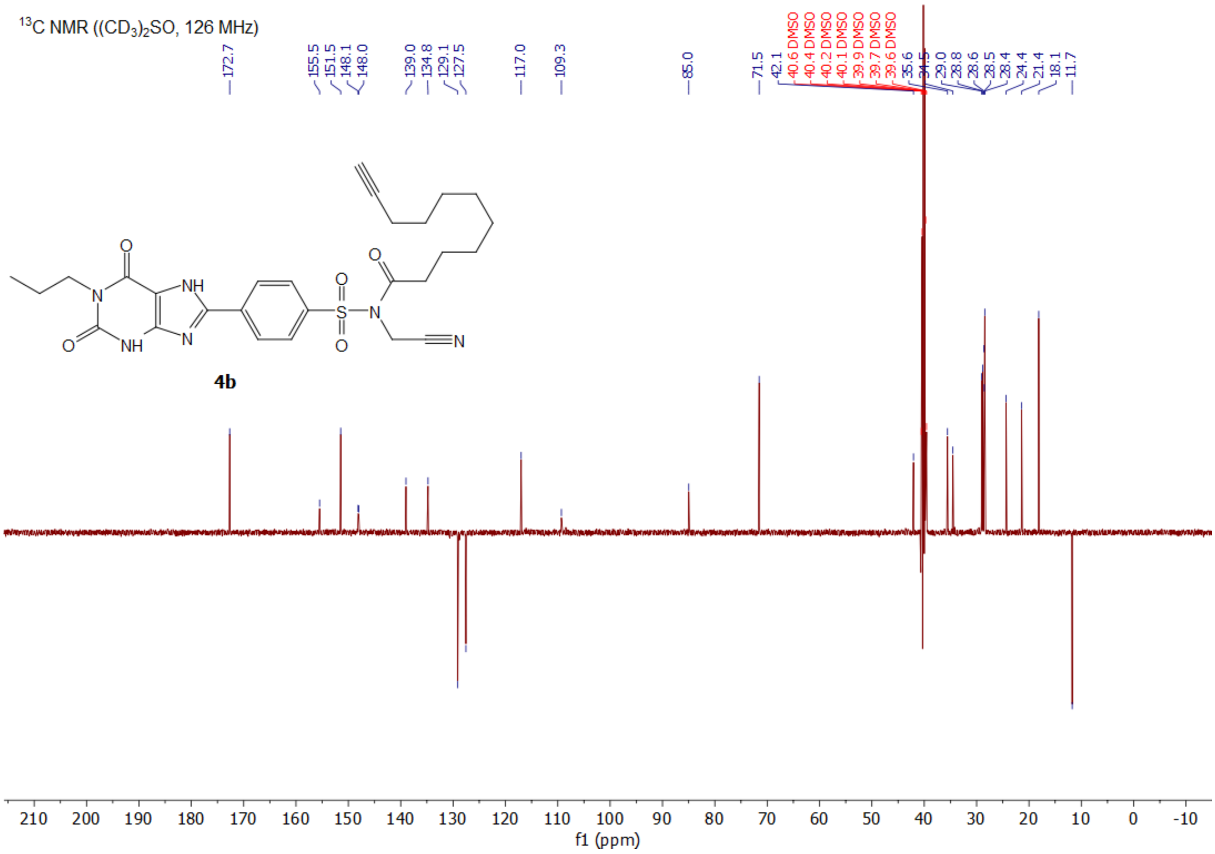

HMBC $((CD_3)_2SO)$

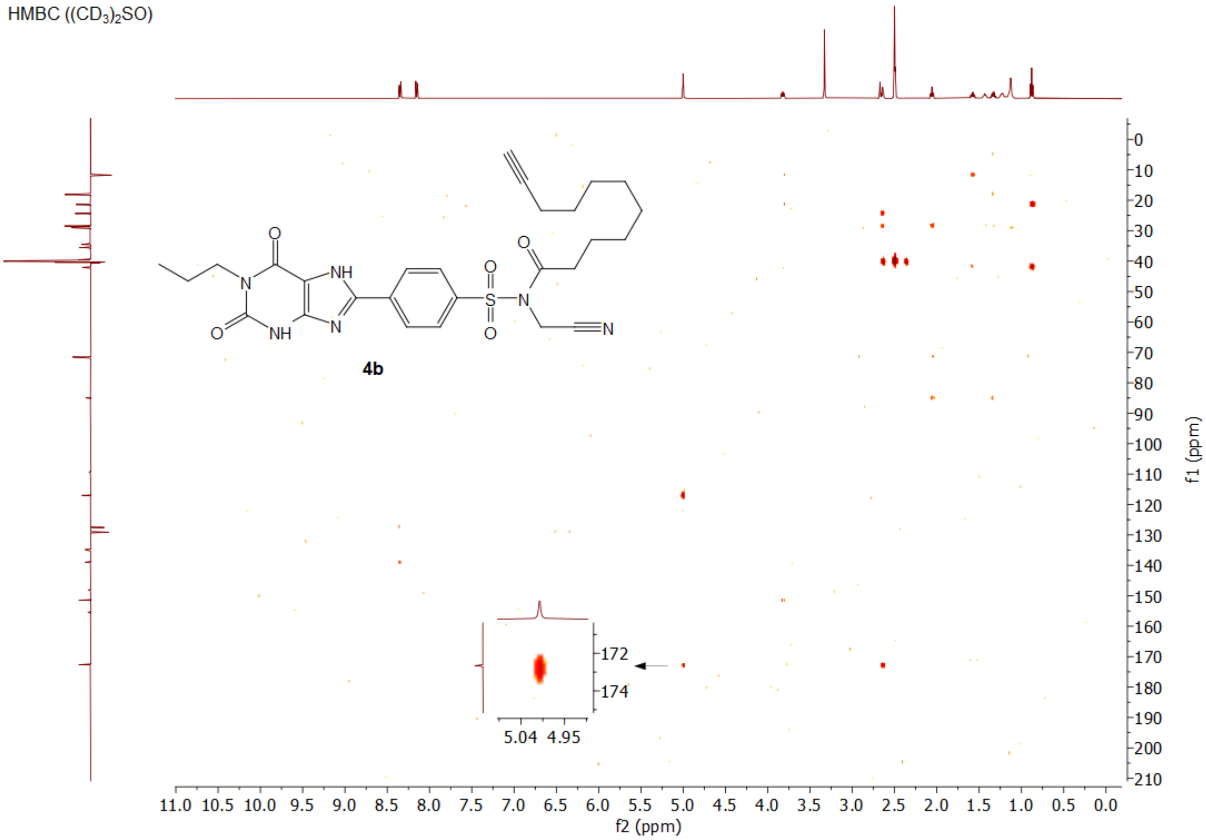

References

(1) Chen, Y.; Zhang, J.; Weng, Y.; Xu, Y.; Lu, W.; Liu, W.; Liu, M.; Hua, T.; Song, G. Cryo-EM Structure of the Human Adenosine A2B Receptor–Gs Signaling Complex. *Sci. Adv.* **2022**, *8* (51), 1–11. https://doi.org/10.1126/sciadv.add3709.

(2) Jespers, W.; Schiedel, A. C.; Heitman, L. H.; Cooke, R. M.; Kleene, L.; van Westen, G. J. P.; Gloriam, D. E.; Müller, C. E.; Sotelo, E.; Gutiérrez-de-Terán, H. Structural Mapping of Adenosine Receptor Mutations: Ligand Binding and Signaling Mechanisms. *Trends Pharmacol. Sci.* **2018**, *39* (1), 75–89. https://doi.org/10.1016/j.tips.2017.11.001.

(3) Beerkens, B. L. H.; Wang, X.; Avgeropoulou, M.; Adistia, L. N.; van Veldhoven, J. P. D.; Jespers, W.; Liu, R.; Heitman, L. H.; IJzerman, A. P.; van der Es, D. Development of Subtype-Selective Covalent Ligands for the Adenosine A2B Receptor by Tuning the Reactive Group. *RSC Med. Chem.* **2022**, *13* (7), 850–856. https://doi.org/10.1039/d2md00132b.

(4) Temirak, A.; Schlegel, J. G.; Voss, J. H.; Vaaßen, V. J.; Vielmuth, C.; Claff, T.; Müller, C. E. Irreversible Antagonists for the Adenosine A2B Receptor. *Molecules* **2022**, *27* (12), 3792. https://doi.org/10.3390/molecules27123792.

(5) Halgren, T. A.; Murphy, R. B.; Friesner, R. A.; Beard, H. S.; Frye, L. L.; Pollard, W. T.; Banks, J. L. Glide: A New Approach for Rapid, Accurate Docking and Scoring. 1. Method and Assessment of Docking Accuracy. *J. Med. Chem.* **2004**, *47* (7), 1750–1759.

(6) Greenwood, J. R.; Calkins, D.; Sullivan, A. P.; Shelley, J. C. Towards the Comprehensive , Rapid , and Accurate Prediction of the Favorable Tautomeric States of Drug-like Molecules in Aqueous Solution. *J. Comput. Aided. Mol. Des.* **2010**, *24* (6), 591–604. https://doi.org/10.1007/s10822-010-9349-1.

(7) The PyMOL Molecular Graphics System, Version 2.5.2. Schrödinger, LLC: New York, NY 2021.

(8) Amelia, T.; Van Veldhoven, J. P. D.; Falsini, M.; Liu, R.; Heitman, L. H.; Van Westen, G. J. P.; Segala, E.; Verdon, G.; Cheng, R. K. Y.; Cooke, R. M.; Van Der Es, D.; Ijzerman, A. P. Crystal Structure and Subsequent Ligand Design of a Nonriboside Partial Agonist Bound to the Adenosine A2AReceptor. *J. Med. Chem.* **2021**, *64* (7), 3827–3842. https://doi.org/10.1021/acs.jmedchem.0c01856.

(9) Yung-Chi, C.; Prusoff, W. H. Relationship between the Inhibition Constant (KI) and the Concentration of Inhibitor Which Causes 50 per Cent Inhibition (I50) of an Enzymatic Reaction. *Biochem. Pharmacol.* **1973**, *22* (23), 3099–3108. https://doi.org/10.1016/0006-2952(73)90196-2.

(10) Kourounakis, A.; Visser, C.; Groote, M. De; Ijzerman, A. P. Differential Effects of the Allosteric Enhancer (2-Amino-4 , 5-Dimethyl- and Antagonist Binding and Function at the Human Wild-Type and a Mutant (T277A) Adenosine A 1 Receptor. *Biochem. Pharmacol.* **2001**, *61* (2), 137–144. https://doi.org/10.1016/S0006-2952(00)00536-0.

(11) Vlachodimou, A.; de Vries, H.; Pasoli, M.; Goudswaard, M.; Kim, S.-A.; Kim, Y.-C.; Scortichini, M.; Marshall, M.; Linden, J.; Heitman, L. H.; Jacobson, K. A.; IJzerman, A. P. Kinetic Profiling and Functional Characterization of 8-Phenylxanthine Derivatives as A2B Adenosine Receptor Antagonists. *Biochem. Pharmacol.* **2022**, *200* (April), 115027. https://doi.org/10.1016/j.bcp.2022.115027.

(12) Rooden, E. J. van; Florea, B. I.; Deng, H.; P, B.-M.; Esbroeck, A. C. M. van; Zhou, J.; Overkleeft, H. S.; Stelt, M. van der. Mapping in Vivo Target Interaction Profiles of Covalent Inhibitors Using Chemical Proteomics with Label-Free Quantification. *Nat. Protoc.* **2018**, *13*, 752–767.

(13) Wessel, D.; Flügge, U. I. A Method for the Quantitative Recovery of Protein in Dilute Solution in the Presence of Detergents and Lipids. *Anal. Biochem.* **1984**, *138* (1), 141–143. https://doi.org/https://doi.org/10.1016/0003-2697(84)90782-6.

(14) Rappsilber, J.; Mann, M.; Ishihama, Y. Protocol for Micro-Purification, Enrichment, Pre-Fractionation and Storage of Peptides for Proteomics Using StageTips. *Nat. Protoc.* **2007**, *2* (8), 1896–1906. https://doi.org/10.1038/nprot.2007.261.

(15) Cox, J.; Mann, M. MaxQuant Enables High Peptide Identification Rates, Individualized p.p.b.-Range Mass Accuracies and Proteome-Wide Protein Quantification. *Nat. Biotechnol.* **2008**, *26* (12), 1367–1372. https://doi.org/10.1038/nbt.1511.

(16) Tyanova, S.; Temu, T.; Sinitcyn, P.; Carlson, A.; Hein, M. Y.; Geiger, T.; Mann, M.; Cox, J. The Perseus Computational Platform for Comprehensive Analysis of (Prote)Omics Data. *Nat. Methods* **2016**, *13* (9), 731–740. https://doi.org/10.1038/nmeth.3901.
